# An insight into differential protein abundance throughout *Leishmania donovani* promastigote growth and differentiation

**DOI:** 10.1007/s10123-022-00259-4

**Published:** 2022-08-05

**Authors:** Pedro J. Alcolea, Ana Alonso, Francisco García-Tabares, Jaime Larraga, Luis T. C. Martins, Franciso J. Loayza, Silvia Ruiz-García, Vicente Larraga

**Affiliations:** 1 Laboratorio de Parasitología Molecular y Vacunas. Unidad de Desarrollo de Fármacos Biológicos, Inmunológicos y Químicos para la Salud Global (BICS). Departamento de Biología Celular y Molecular, Centro de Investigaciones Biológicas Margarita Salas, Consejo Superior de Investigaciones Científicas (CIBMS-CSIC), Calle Ramiro de Maeztu 9, 28040 Madrid, Spain; 2 Servicio de Proteómica y Genómica, Centro de Investigaciones Biológicas Margarita Salas, Consejo Superior de Investigaciones Científicas (CIBMS-CSIC), Calle Ramiro de Maeztu 9, 28040 Madrid, Spain

**Keywords:** *Leishmania donovani*, Promastigotes, Protein levels, Stress, Antigens

## Abstract

*Leishmania donovani* causes anthroponotic visceral leishmaniasis, responsible for about 50,000 annual deaths worldwide. Current therapies have considerable side effects. Drug resistance has been reported and no vaccine is available nowadays. The development of undifferentiated promastigotes in the sand fly vector’s gut leads to the promastigote form that is highly infective to the mammalian host. Fully differentiated promastigotes play a crucial role in the initial stages of mammalian host infection before internalization in the host phagocytic cell. Therefore, the study of protein levels in the promastigote stage is relevant for disease control, and proteomics analysis is an ideal source of vaccine candidate discovery. This study aims to get insight into the protein levels during the differentiation process of promastigotes by 2DE-MALDI-TOF/TOF. This partial proteome analysis has led to the identification of 75 proteins increased in at least one of the *L. donovani* promastigote differentiation and growth phases. This study has revealed the differential abundance of said proteins during growth and differentiation. According to previous studies, some are directly involved in parasite survival or are immunostimulatory. The parasite survival–related proteins are ascorbate peroxidase; cystathionine β synthase; an elongation factor 1β paralog; elongation factor 2; endoribonuclease L-PSP; an iron superoxide dismutase paralog; GDP-mannose pyrophosphorylase; several heat shock proteins—HSP70, HSP83-17, mHSP70-rel, HSP110; methylthioadenosine phosphorylase; two thiol-dependent reductase 1 paralogs; transitional endoplasmic reticulum ATPase; and the AhpC thioredoxin paralog. The confirmed immunostimulatory proteins are the heat shock proteins, enolase, and protein kinase C receptor analog. The potential immunostimulatory molecules according to findings in patogenic bacteria are fructose-1,6-diphophate aldolase, dihydrolipoamide acetyltransferase, isocitrate dehydrogenase, pyruvate dehydrogenase E1α and E1β subunits, and triosephosphate isomerase. These proteins may become disease control candidates through future intra-vector control methods or vaccines.

**Supplementary Information:**

The online version contains supplementary material available at 10.1007/s10123-022-00259-4.

## Introduction

*Leishmania donovani* (Kinetoplastida: Trypanosomatidae) is the causative agent of anthroponotic visceral leishmaniasis (AVL), also named Kala-Azar or dumdum fever. AVL is fatal if left untreated (Desjeux 2001; WHO 2010). The estimated disease burden of visceral leishmaniasis (VL) is 500,000 cases, including AVL and zoonotic visceral leishmaniasis (ZVL). VL causes approximately 50,000 annual deaths (WHO 2010; Alvar et al. 2012; Desjeux 2004). East Africa and the Indian subcontinent are the main *L. donovani* endemic areas. Vaccines are not available, and the approved drugs frequently cause adverse effects and resistance.

The *Leishmania* spp. life cycle is digenetic. The promastigote stage is extracellular and undergoes differentiation in the sandfly vector (Psychodidae: Phlebotominae) gut, leading to highly infective forms called metacyclic promastigotes. The sandfly injects mature promastigotes into the mammalian host’s dermis during bloodmeal intakes. Most promastigotes are killed in about 3 min by the complement system (Moreno et al. 2007; Dominguez et al. 2002), and only a fraction of the population enters phagocytic host cells, where they rapidly differentiate into the amastigote stage. Amastigotes survive within the phagolysosome's acidic microenvironment in the presence of hydrolytic enzymes (Zilberstein et al. 2008; Zilberstein and Shapira 1994) at 32–37 °C, depending on the species. Regarding this sequence of events during the life cycle, fully differentiated promastigotes likely bear most vaccine candidates (Alcolea et al. 2016a), and amastigotes most drug targets. However, the opposite should not be discarded in vaccine development as the objective is to block both stages.

High-throughput quantitative transcriptomic and proteomic approaches allowed for differential gene expression analysis promastigote-to-amastigote development (Lahav et al. 2011; Rosenzweig et al. 2008a, 2008b; Saxena et al. 2007; Srividya et al. 2007), but not *L. donovani* promastigote growth and differentiation. In this study, protein levels in the early logarithmic, mid-logarithmic, and stationary phase of *L. donovani* promastigotes have been compared employing two-dimensional electrophoresis (2DE). Therefore, the promastigote growth and differentiation process leading to an infectivity increase has been compared in terms of relative protein abundance. Protein identification was performed by MALDI-TOF/TOF mass spectrometry. Interestingly, some differentially abundant proteins are immunostimulatory or are involved in parasite survival, according to previous studies.

## Materials and methods

### Promastigote cultures

Promastigotes of the *L. donovani* Karthoum strain (MHOM/SD/43/124) were kindly provided by A. Toraño and M. Domínguez (Department of Immunology, Centro Nacional de Microbiología, Virología e Inmunología Sanitarias, Instituto de Salud Carlos III, Majadahonda, Spain). Three cultures were set at an initial cell density of 2 × 10^6^/ml in RPMI 1640 supplemented with L-glutamine (Life Technologies, Carlsbad, CA), 10% heat-inactivated fetal bovine serum (Lonza, Basel, Switzerland), and 100 μg/ml streptomycin—100 IU/ml penicillin (Life Technologies), and incubated at 27 °C. Cell density was estimated with a Neubauer chamber and total protein extracts from 10^8^ promastigotes per culture were prepared daily after harvesting them at 2000 *g* for 10 min.

### Protein extracts

Samples containing 10^8^ promastigotes were washed with PBS and lysed with 150 μl of a buffer containing 8.4 M urea, 2.4 M thiourea, 5% CHAPS, 50 mM DTT, 1% Triton X-100, and 50 μg/ml DNase I. The buffer also contained Mini EDTA-free Protease Inhibitor Cocktail at the amount specified by the manufacturer (Roche, Mannheim, Germany). Lysis was allowed for 30 min under mild rotation. Then, the extracts were centrifuged at 8000 *g* for 10 min. The supernatants were precipitated with methanol/chloroform (Wessel and Flugge 1984). Once the sediments dried, they were resuspended in 2 × rehydration buffer (7 M urea, 2 M thiourea, 4% CHAPS, 0.003% bromophenol blue). Protein concentration was measured with the *RC DC protein assay kit* (BioRad, Hercules, CA) according to the manufacturer’s instructions, and by densitometry of SDS-PAGE runs (Alcolea et al. 2011).

### 2DE analysis of the relative protein levels

The input for 2DE was 50 μg per sample, which were equivalent to ~ 3 × 10^7^ promastigotes. After diluting to 140 μl with 2 × isoelectrofocusing (IEF) buffer (18.2 mM DTT and 0.5% *IPG buffer solution* pH 3–10, BioRad), IEF runs were carried out in a Protean IEF Cell system (BioRad) using IPG strips of 7 cm in length, and a non-linear gradient from pH 3 to 10 (BioRad). More than 12,000 V·h were reached in all steps, which were: 50 V for 12 h; 250 V for 1 h; 500 V for 1 h; 1000 V for 1 h; 2000 V for 1 h; linear ramp to 8000 V for 1 h; and 8000 V up to 3500 V·h. Then, the proteins were separated by 12% SDS-PAGE in a pre-cooled MiniProtean 3 Dodeca Cell system (BioRad) at 0.5 W/gel for the first 30 min and 1.5 W for 5 min more than required for the dye front to reach the bottom edge. After staining with SYPRO Ruby (BioRad), the images were analyzed with PDQuest 2D Advanced 8.0.1 software (BioRad) according to the manufacturer’s instructions. Briefly, the images of all experimental conditions and replicates were overlapped, generating a composite image (i.e., master gel). Then, spots were automatically detected in all individual member gel images and in the master gel, and undetected spots were manually set. The landmark tool allowed enhancing the accuracy of pairwise spot recognition across gels. Then, a manual check of all spot histograms and 3D density graphs was carried out in order to remove artifacts. The normalization method was based on the *Total Quantity in Valid Spots* algorithm. The reference samples for calculation of the gene expression ratios were early logarithmic phase promastigotes (day 2).

### MALDI-TOF/TOF mass spectrometry protein identification

Spot excision from 2DE gels was carried out with EXQuest Spot Cutter (BioRad). In-gel digestion was carried out with porcine trypsin, and peptides were prepared for MALDI-TOF/TOF mass spectrometry as described (Alcolea et al. 2011). First, the spots were washed with 50 mM ammonium bicarbonate. Then, they were washed with acetonitrile, reduced with 10 mM DTT in the presence of 25 mM ammonium bicarbonate at 56 °C for 20 min, and alkylated with 50 mM iodoacetamide in a 50 mM ammonium bicarbonate solution. The wash steps were repeated and the spots were dried at 40 °C. Protein digestions were performed with 16 ng/ml modified porcine trypsin (Promega, Madison, WI) in a 25% acetonitrile-50 mM ammonium bicarbonate solution at 37 °C for 6 h. The reactions were stopped by addition of 0.5% (v/v) trifluoroacetic acid (TFA). The peptides were extracted with TFA for 15 min. The eluted tryptic peptides were dried in a vacuum centrifuge and resuspended in 4 ml of a 30%/15%/0.1% water/isopropanol/TFA solution. The 0.8 μl digests were mixed with 0.8 μl aliquots of 3 μg/μl α-cyano-4-hydroxycinnamic acid (Sigma), organized in an OptiTOF™ Plate (Life Technologies), and air-dried at room temperature. The ABI 4800 MALDI-TOF/TOF mass spectrometer (Life Technologies) runs were performed in positive reflector mode at 25 kV for MS and 1 kV for MS/MS. Using ABI 4000 Series Explorer Software 3.6 (Life Technologies), peptide mass fingerprinting (PMF) and MS/MS fragment ion spectra were smoothed and corrected to zero baseline. Each PMF spectrum was internally calibrated with the mass signals of ions generated in trypsin autolysis in order to reach < 25 ppm accuracy in mass measurements. Trypsin and keratin mass signals and potential sodium and potassium adducts (+ 21 Da and + 39 Da) were removed from the peak list.

### Statistical analysis and meta-analysis

Three biological replicates of the experiment were performed. After spot identification and curation (“2DE analysis of the relative protein levels”), the relative expression levels were calculated with PD Quest 2D Advanced 8.0.1 software (BioRad). Spots showing statistically significant (Student’s *t*-test, *α* > 0.05) differences in protein levels (≥ 1.7-fold) were selected.

The PMF and fragment ion spectra were processed with MASCOT 2.1 and Global Protein Server Explorer 4.9 (Life Technologies) for protein identification with the following settings: trypsin as the enzyme for peptide generation; allowed one missed cleavage; carbamidomethyl cystein as fixed modification due to treatment with iodoacetamide; oxidation of methionine as a variable modification; ± 50 ppm mass tolerance for precursors, and ± 0.3 Da for MS/MS fragment ions; and confidence interval for protein identification ≥ 95% (*p* < 0.05). Only peptides with an individual ion score above the identity threshold (52) were considered correctly identified. The last release (TriTrypDB rel. 52, 2021; www.tritrypdb.org) of the resequenced and the *de novo* assembled *L. infantum* JPCM5 genome (Gonzalez-de la Fuente et al. 2017) was used as the reference sequence for protein identifications because this genome sequence has been extensively curated for almost 15 years and because *L. infantum* belongs to the *L. donovani* species complex. Gene Ontology (GO) enrichment analysis was performed with REVIGO (Supek et al. 2011). Interactive graphs of GO Biological Process (GOBP) and GO Molecular Function (GOMF) terms were generated for the protein sets increased and decreased on days 4 and 6 with respect to day 2.

### Western blot

Twenty micrograms of each protein extract were separated by 10% SDS-PAGE at 12 mA for 30 min and 30 mA for 90 min. Semi-dry electrotransfer onto Trans-Blot® 0.2 μm nitrocellulose membranes was performed at 1.3A and 25 V for 10 min using the Trans-Blot® Turbo Transfer System (BioRad). Next, the membranes were blocked with a 5% skimmed milk solution in PBS with 0.1% Tween 20 (Sigma) for 1 h at room temperature and washed three times with PBS containing 0.1% Tween 20 (PBS-Tween) for 15, 5, and 5 min, respectively. Thereafter, the membranes were incubated with 1∶1000-diluted rabbit anti-LACK polyclonal serum in the blocking solution for 2 h (Alcolea et al. 2016b). The membrane was washed before and after incubation with 1∶2,000 HRP-conjugated goat anti-rabbit IgG (DAKO, Ely, UK) for 1.5 h, and developed with ECL reagents (GE Healthcare, Pittsburg, PA) in a Chemi-Doc™ (BioRad) instrument following the manufacturers’ instructions. The antibodies were removed from the membrane by a 1 h stripping step at 50 °C in a solution containing 111 mM β-mercaptoethanol, 347 mM SDS, and 62.5 mM Tris–HCl pH6.8. During this step, thorough mixing was performed for 10 s every 5 min. The hybridization procedure was repeated using a 1:10,000 dilution of a monoclonal mouse anti-*L. mexicana* gGAPDH antibody kindly provided by Paul Michels (University of Edinburgh), which was the loading control (Alcolea et al. 2014).

## Results and discussion

### Analysis of protein levels in *L. donovani* promastigotes by 2DE-MALDI-TOF/TOF

**Fig. 1:**
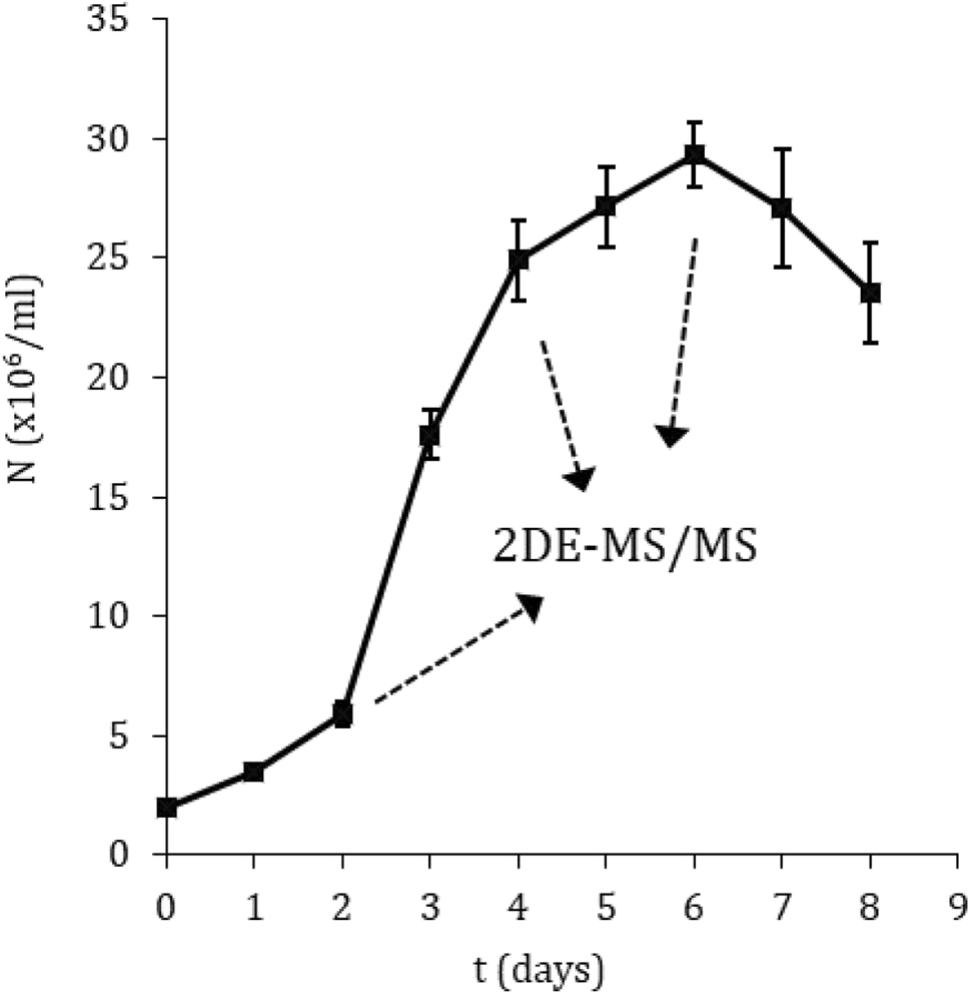
Growth kinetics of *L. donovani* promastigotes and experimental design. Promastigote samples were obtained on day 2 (early logarithmic phase), day 4 (mid-logarithmic phase), and day 6 (stationary phase). These samples were analyzed by 2DE-MALDI-TOF/TOF analysis. Three biological replicates were performed

**Fig. 2:**
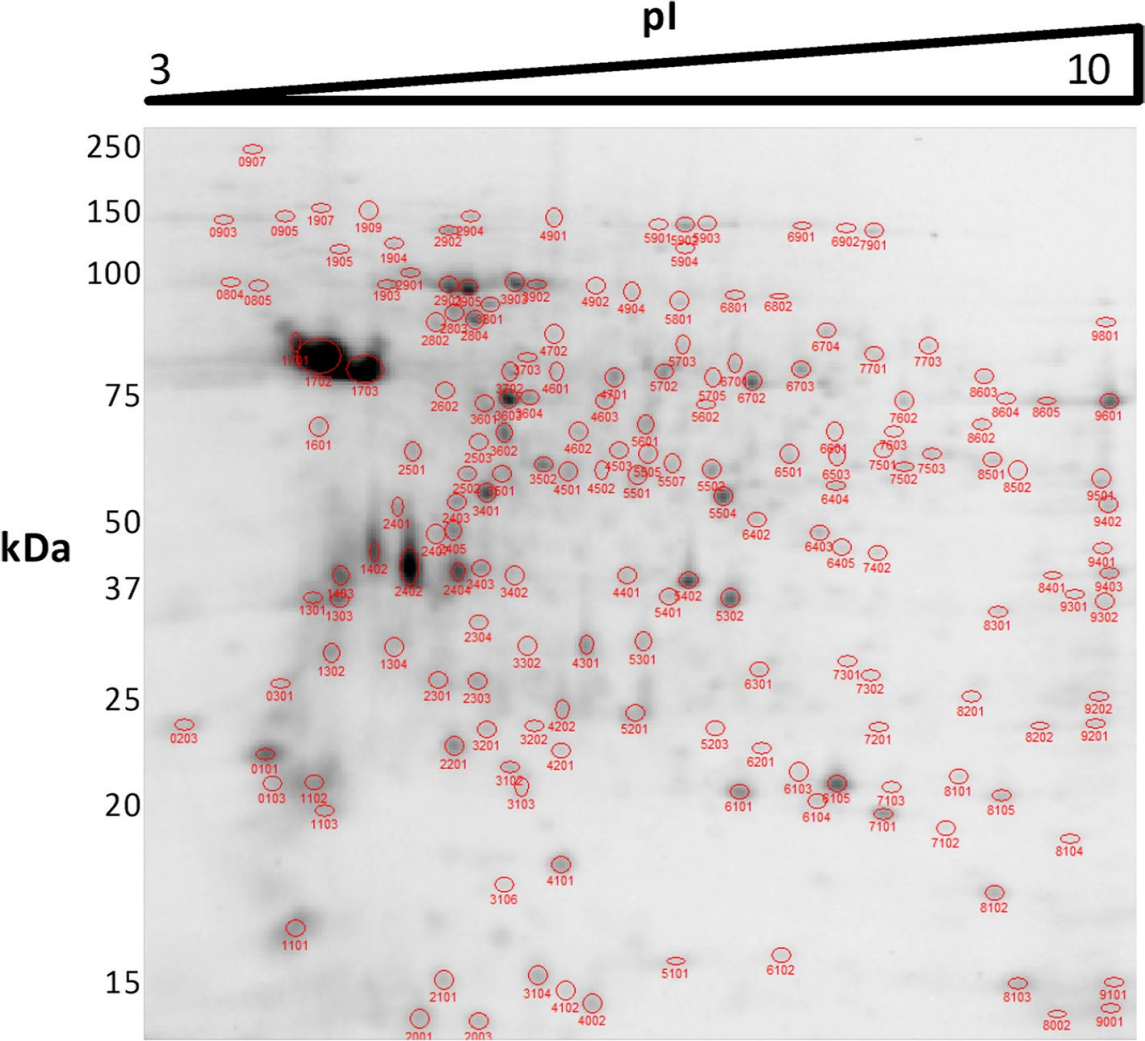
2DE of total protein extracts from *L. donovani* promastigotes in early logarithmic phase (day 2). IEF was performed in a non-linear 3–10 pH interval. The 2DE gel represents one out of three biological replicates and was stained with SYPRO Ruby. The image was analyzed with PDQuest 2D Advanced 8.0.1 software together with all others, generating a master gel composite image. Most spots were automatically recognized. All spots were manually curated. The spots that were not automatically recognized were manually curated and included in analysis when the 3D intensity graph showed a 3D gaussian, Poisson, hypergeometric, or bimodal distribution. Those showing an irregular intensity distribution were considered noise and were not included in analysis. The spots showing this pattern that had been automatically recognized by the software were removed from analysis. 176 spots were recognized in this gel as a result of the manual curation process

**Fig. 3:**
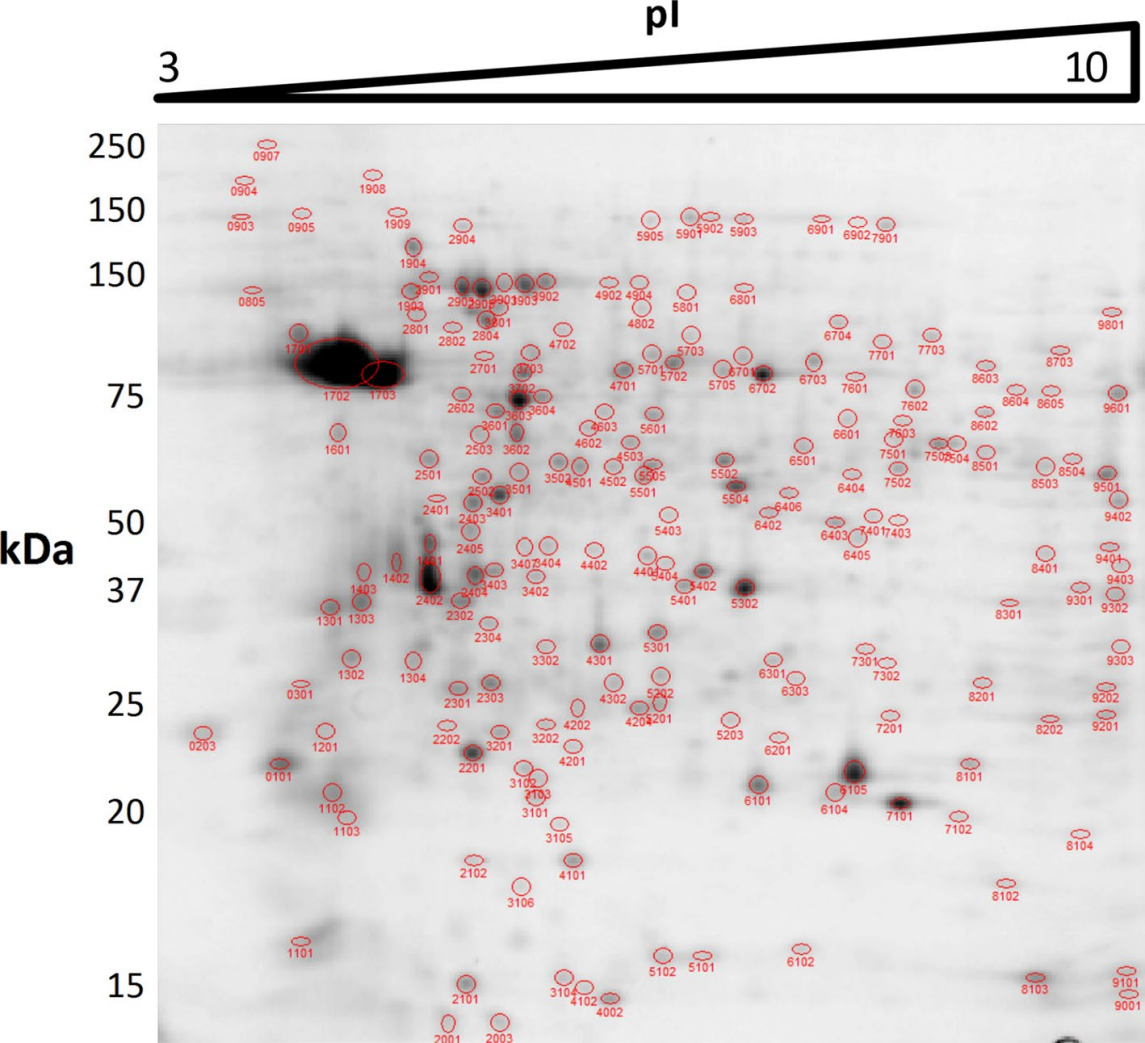
2DE of total protein extracts *L. donovani* promastigote in mid-logarithmic phase (day 4). IEF was performed in a non-linear 3–10 pH interval. The 2DE gel represents one out of three biological replicates and was stained with SYPRO Ruby. The image was analyzed with PDQuest 2D Advanced 8.0.1 software together with all others, generating a master gel composite image. Most spots were automatically recognized. All spots were manually curated. The spots that were not automatically recognized were manually curated and included in analysis when the 3D intensity graph showed a 3D gaussian, Poisson, hypergeometric, or bimodal distribution. Those showing an irregular intensity distribution were considered noise and were not included in analysis. The spots showing this pattern that had been automatically recognized by the software were removed from analysis. 205 spots were recognized in this gel as a result of the manual curation process

**Fig. 4:**
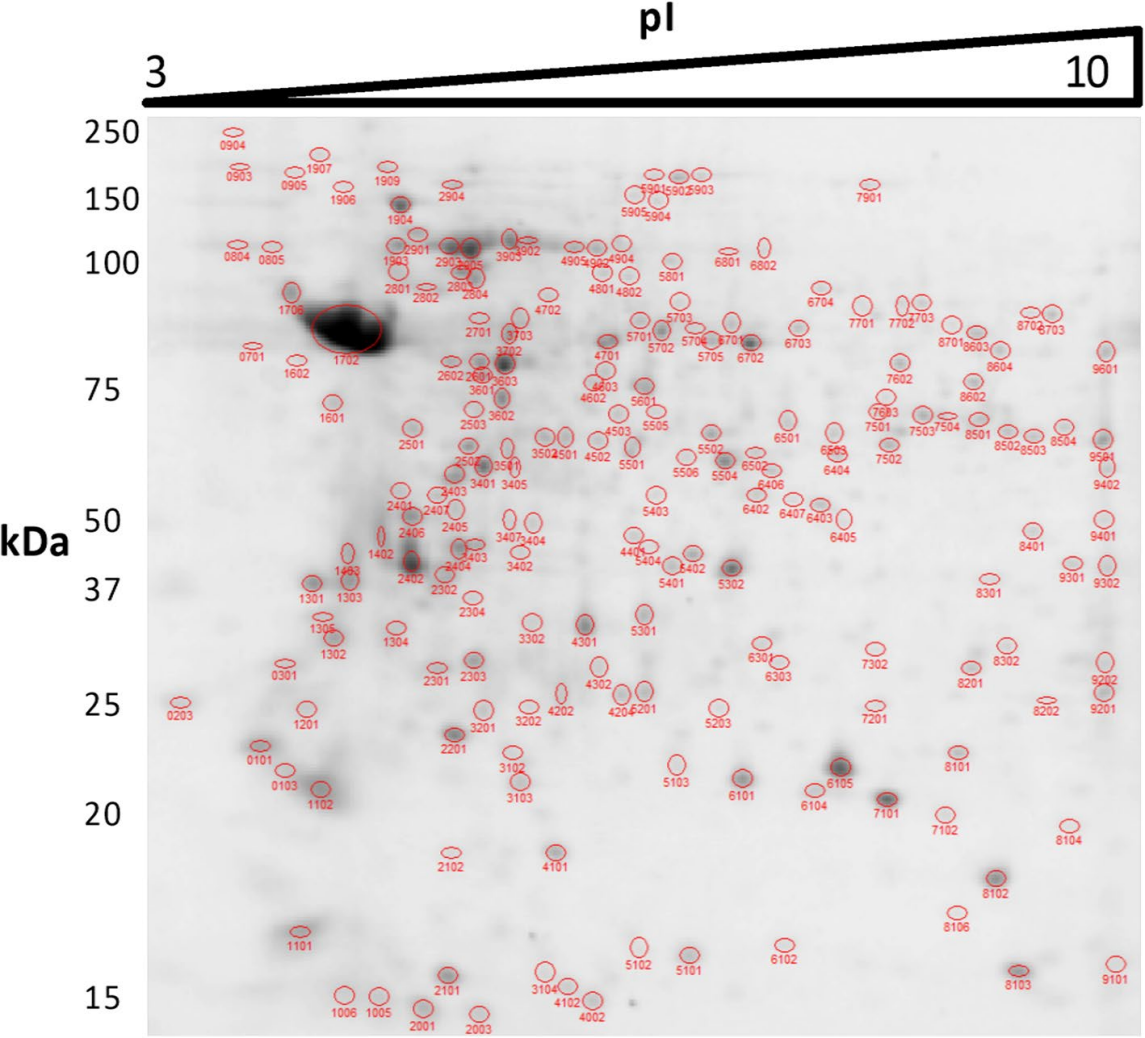
2DE of total protein extracts from *L. donovani* promastigotes in stationary phase (day 6). IEF was performed in a non-linear 3–10 pH interval. The 2DE gel represents one out of three biological replicates and was stained with SYPRO Ruby. The image was analyzed with PDQuest 2D Advanced 8.0.1 software together with all others, generating a master gel composite image. Most spots were automatically recognized. All spots were manually curated. The spots that were not automatically recognized were manually curated and included in analysis when the 3D intensity graph showed a 3D gaussian, Poisson, hypergeometric, or bimodal distribution. Those showing an irregular intensity distribution were considered noise and were not included in analysis. The spots showing this pattern that had been automatically recognized by the software were removed from analysis. 204 spots were recognized in this gel as a result of the manual curation process

Protein extracts were prepared from promastigote samples at the following logistic growth curve time points (Fig. 1): day 2 (early logarithmic phase), day 4 (mid-logarithmic phase), and day 6 (stationary phase). The proteomics experiment was performed in triplicate including analysis of biological variation in the experimental design (i.e., individual processing of three independent cultures). One out of three replicate 2DE gels per condition is shown in Figs. 2, 3, and 4.

**Fig. 5:**
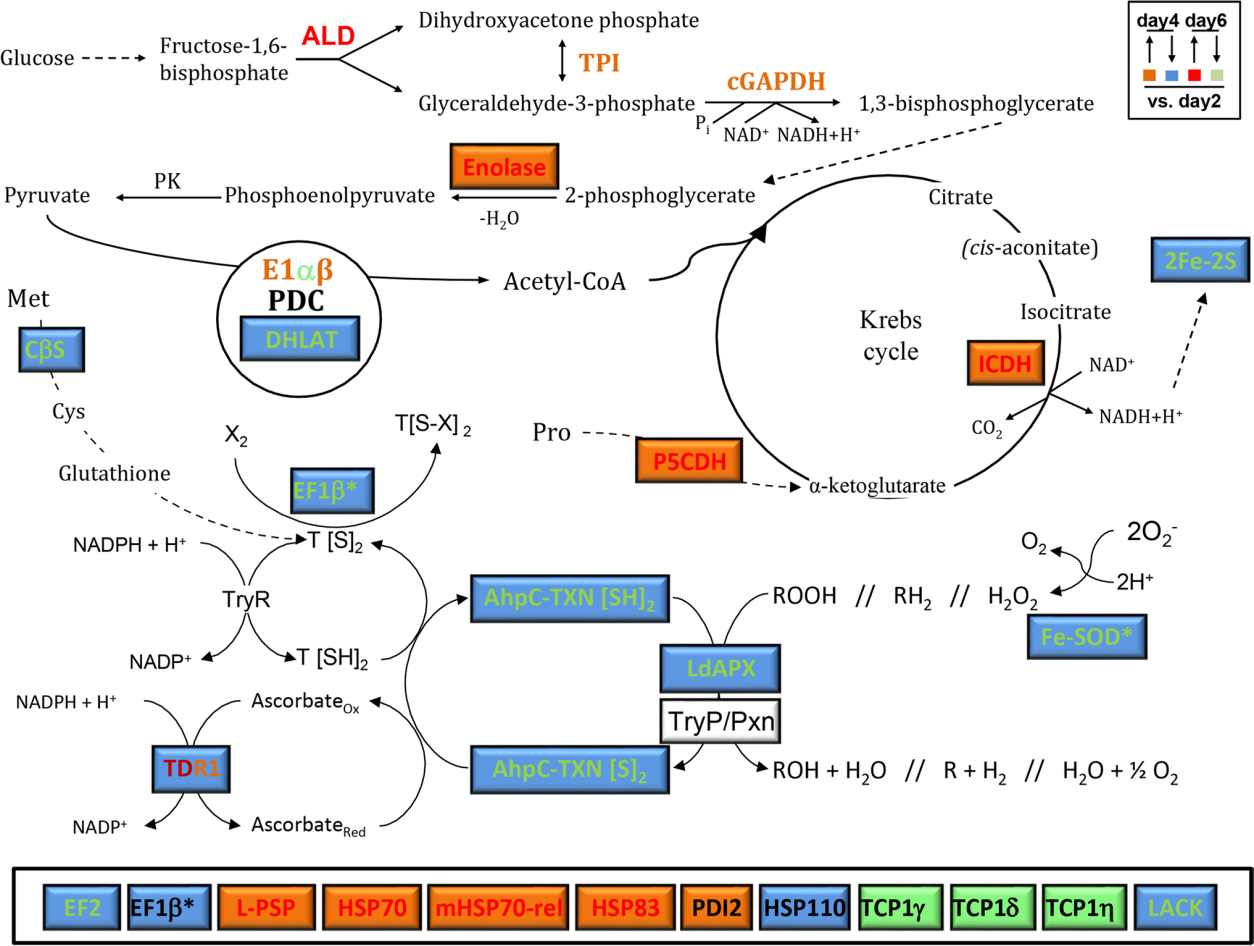
Differential abundance of potential immunostimulatory proteins and drug targets in *L. donovani* promastigotes. The color legend indicates upregulation (↑) and downregulation (↓) at days 4 (mid-logarithmic phase) and 6 (stationary phase) compared to day 2 (early logarithmic phase)

Statistical analysis included normalization with the *Total Quantity in Valid Spots* algorithm and statistical inference using the paired Student’s *t*-test (threshold values: ≥ 1.7-fold expression; *p* < 0.05). For this purpose, PDQuest software (BioRad) was used. The number of differentially expressed proteins with respect to day 2 promastigotes (MASCOT score > 52) that were identified in at least one of the conditions studied is 75 (Table 1). About 200 quality spots could be confirmed in each 2DE gel. The total number of spots containing differentially abundant proteins was 81, but 6 could not be identified by MALDI-TOF/TOF. Some of the differentially abundant proteins found (Table 1) were previously described as potential immunostimulatory proteins and some others are related to stress (Fig. 5).

**Table 1:** Differentially abundant proteins in *L. donovani* promastigotes. Spot numbers, TriTrypDB identifiers, protein names, MASCOT scores (significant over 52) and gene expression ratios referred to day 2 are provided

Spot	Accesion no	Product name	MASCOT score	Day 4	Day 6
LdK.0103	LINF_130008200-500	α-tubulin	381	7.3	1.9
LdK.0104	LINF_270008400	Hypothetical protein, conserved	62	3.2	2.3
LdK.0301	LINF_080017700	β-tubulin (fragment)	325	n.d	3.2
LdK.0701	LINF_310033400	Ferredoxin, 2Fe-2S-like protein	71	0.5	0.3
LdK.0903	LINF_130009200/ LINF_360007000	Flagellar radial spoke protein, putative/elongation factor 2	219/214	2.4	n.d
LdK.1101	LINF_130010600/ LINF_140014400	40S ribosomal protein S12, putative/calpain-like cysteine peptidase, putative	221/150	0.5	n.d
LdK.1102	LINF_130008200-500	α-tubulin	75	n.d	2.5
LdK.1201	LINF_140006800	AhpC/TSA family/thioredoxin-like-putative	312	0.3	0.3
LdK.1302	LINF_360040400	14-3-3 protein 1, putative	1250	n.d	2.2
LdK.1303	LINF_080017700	β-tubulin	391	2.6	3.4
LdK.1305	LINF_080017700/LINF_080017700	β-tubulin	214/124	n.d	126.5
LdK.1402	LINF_280029400	Glycosomal membrane protein, putative	80	0.6	n.d
LdK.1403	LINF_080017700	β-tubulin	174	0.4	n.d
LdK.1602	LINF_130008100-600	α-tubulin	517	n.d	0.2
LdK.1903	LINF_260017400	Heat shock protein 70-related protein, mitochondrial precursor, putative	946	n.d	1.7
LdK.1904	LINF_330009400-500	Heat shock protein 83-17	639/639	n.d	2.1
LdK.1905	LINF_340007000	Domain of unknown function (DUF1935), putative	113	1.7	n.d
LdK.2102	LINF_130008100-600	α-tubulin	316	1.8	n.d
LdK.2302	LINF_130008100-600	α-tubulin	517	2.1	n.d
LdK.2401	LINF_270020600	60S acidic ribosomal protein P0, putative	139	0.4	n.d
LdK.2402	LINF_180016900	Hypothetical protein, conserved	72	n.d	1.7
LdK.2403	LINF_250023800	Pyruvate dehydrogenase E1 beta subunit, putative	588	2.3	n.d
LdK.2404	LINF_340014000	Elongation factor 1-beta	157	0.5	n.d
LdK.2405	LINF_270006800	Proteasome alpha 7 subunit, putative	243	n.d	0.6
LdK.2406	LdBPK_251510.1	Peptide chain release factor 1, mitochondrial, putative (RF1)	64	0.3	n.d
LdK.2503	LINF_230007100	Endoribonuclease L-PSP (pb5), putative	241	3.2	n.d
LdK.2602	LINF_140018000	Enolase	582	2.3	2.4
LdK.2701	LINF_130008100-600	α-tubulin	429	0.4	0.3
LdK.2801	LINF_170013800	VID27 cytoplasmic protein, putative	303	2.3	4.7
LdK.2802	LINF_330007600	Thiol-dependent reductase 1	317	3	2.3
LdK.2901	LINF_280017800	Heat shock 70-related protein 1-mitochondrial precursor, putative	621	n.d	1.8
LdK.2902	LINF_360019800	Transitional endoplasmic reticulum ATPase, putative	311	5.8	n.d
LdK.2904	LINF_180019100	Heat shock protein 110, putative	447	0.4	n.d
LdK.2905	LINF_280035900-6000	HSP70	877	1.8	2.9
LdK.3104	LINF_070006400	Hypothetical protein, conserved	61	n.d	0.3
LdK.3302	LINF_270006800	Proteasome alpha 7 subunit, putative	206	n.d	1.7
LdK.3303	LINF_250027000	2–4-dihydroxyhept-2-ene-1-7-dioic acid aldolase-putative	149	n.d	1.9
LdK.3405	LINF_360081500	Protein disulfide isomerase 2 (PDI2)	147	2.1	n.d
LdK.3407	LINF_140018000	Enolase	755	n.d	36.6
LdK.3702	LINF_360072600	Carboxypeptidase, putative	624	2.2	0.5
LdK.3703	LINF_130005800	Carboxypeptidase, putative	428	7	n.d
LdK.3801	LINF_130008100-600/LINF_360027200	α-tubulin/Chaperonin HSP60, mitochondrial precursor	249/183	2.6	0
LdK.3901	LINF_280035900-6000	HSP70	826	1.7	n.d
LdK.3903	LINF_300030200	Heat shock 70-related protein 1, mitochondrial precursor, putative	705	2.6	n.d
LdK.4202	LINF_320017000	Hypothetical protein, conserved	71	0.5	n.d
LdK.4503	LINF_170008800	Cystathionine β-synthase (CβS)	219	n.d	1.7
LdK.4601	LINF_200019300	Paralyzed flagella protein 16, putative	199	16.9	n.d
LdK.4902	LINF_280035900-6000	HSP70	97	3.4	2.1
LdK.5102	LINF_230007100	Endoribonuclease L-PSP (pb5), putative	233	n.d	38.6
LdK.5402	LINF_280034700-800	Receptor for activated C kinase 1 (LACK)	645	0.6	0.4
LdK.5404	LINF_280034700-800	Receptor for activated C kinase 1 (LACK)	273	0.3	0.4
LdK.5501	LINF_230006100	GDP-mannose pyrophosphorylase	436	2.1	n.d
LdK.5601	LINF_010012800/LINF_210024300	Eukaryotic initiation factor 4a, putative/ATP-dependent RNA helicase SUB2, putative	508/412	n.d	1.7
LdK.5602	LINF_280024500	Replication factor A, 51 kDa subunit, putative	418	n.d	0
LdK.5701	LINF_360034400	Dihydrolipoamide acetyltransferase precursor, putative	513	0.5	0.3
LdK.5703	LINF_230021100	T-complex protein 1, gamma subunit, putative	427	n.d	0.3
LdK.5902	LINF_360007000-100	Elongation factor 2	610	0.6	0.5
LdK.5903	LINF_360007000-100	Elongation factor 2	559	0.4	0.4
LdK.6403	LINF_050013500	Methylthioadenosine phosphorylase, putative	505	0.6	n.d
LdK.6502	LINF_250022600	Hypothetical protein, conserved	388	0.5	n.d
LdK.6503	LINF_210024400	Hypothetical protein, conserved	174	n.d	1.8
LdK7101	LINF_320033200	Iron superoxide dismutase, putative (Fe-SOD)	659	2.8	2.0
LdK.7302	LINF_340005600	Ascorbate peroxidase, putative (APX)	189	0.6	0.6
LdK.7402	LINF_360018400	Fructose-1,6-bisphosphate aldolase	313	n.d	2.1
LdK.7502	LINF_350012900	Aspartate aminotransferase, putative	728	0.3	0.5
LdK.7701	LINF_210018500	T-complex protein 1, delta subunit, putative	501	n.d	0.5
LdK.7703	LINF_350044000	T-complex protein 1, eta subunit, putative	570	n.d	0.6
LdK.8001	LINF_110017900/LINF_290025400	40S ribosomal protein S15A, putative/40S ribosomal protein S15A, putative	135/135	0.2	0
LdK.8202	LINF_240013700	Triosephosphate isomerase	106	1.8	n.d
LdK.8401	LINF_360030600	Glyceraldehyde 3-phosphate dehydrogenase, cytosolic	234	2.2	n.d
LdK.8502	LINF_100008300	Isocitrate dehydrogenase [NADP], mitochondrial precursor, putative	316	1.7	1.9
LdK.8503	LINF_180019200	Pyruvate dehydrogenase E1 component alpha subunit, putative	268	n.d	0.5
LdK.8702	LINF_240012800/LINF_030006800	Malic enzyme, putative/δ-1-pyrroline-5-carboxylate dehydrogenase, putative	243/219	2.4	n.d
LdK.8703	LINF_030006800	δ-1-pyrroline-5-carboxylate dehydrogenase, putative	763	10.5	11.7
LdK.9501	LINF_360018400	Fructose-1,6-bisphosphate aldolase	487	n.d	0.5

### The LACK levels decrease throughout *L. donovani* promastigote growth

The activated protein kinase C receptor analog (LACK) abundance peak is reached at the beginning of *L. donovani* promastigote growth and differentiation. As these processes progress, a considerable gradual decrease is observed (Table 1), which has been validated by Western blot (Fig. 5; Supplementary Information (SI): Fig. 11). A similar expression pattern has been observed in *L. infantum* (Alcolea et al. 2011), *L. amazonensis* (Alcolea et al. 2016b), and the non-pathogenic trypanosomatid *Crithidia fasciculata*, which contains the CACK ortholog (Alcolea et al. 2014). The steady-state transcript levels are not coincident with the protein levels because the *L. infantum* LACK transcript levels are constant (Gonzalez-Aseguinolaza et al. 1999; Alcolea et al. 2010) thanks to translational or post-translational regulation. *L. amazonensis* causes ACL, and *L. infantum* and *L. donovani* cause VL, although both species have specific features leading to different disease transmission and progression. *L. donovani* causes AVL, whereas *L. infantum* VL is zoonotic (ZVL), of which canids are the main reservoirs. ZVL in humans is especially prevalent in children and immunosuppressed patients (Cruz et al. 2006; Pasquau et al. 2005). However, an outbreak involving lagomorphs as reservoirs remains active in Spain (Arce et al. 2013; Jimenez et al. 2014; Molina et al. 2012). LACK is an antigenic protein able to partially protect against *L. infantum* in dogs (Ramiro et al. 2003; Ramos et al. 2008, 2009; Alcolea et al. 2019a). The *L. infantum* LACK protein localizes in the cytoplasm particulate fraction near the plasma membrane. The LACK levels decrease throughout promastigote growth. However, they are sufficiently high in the stationary phase. A LACK-based DNA vaccine against canine leishmaniasis has been achieved (Ramiro et al. 2003; Ramos et al. 2008, 2009; Alcolea et al. 2019a). The LACK gene expression regulation behavior is similar in *L. donovani* and *L. amazonensis* suggesting that the protein is also protective in these species.

### Differential abundance of parasite survival and immunostimulatory proteins

**Fig. 6:**
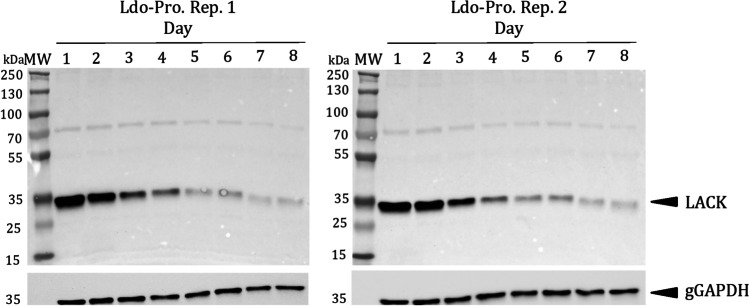
The LACK levels decrease during *L. donovani* promastigote growth and differentiation. Detection and analysis of LACK relative abundance by Western blot in 10 μg total protein extracts using 1:1000-diluted rabbit anti-LACK polyclonal antibody. The reference protein is gGAPDH, which was detected with a specific monoclonal antibody at a 1:10,000 dilution

Some differentially abundant proteins from Table 1 are involved in parasite survival or are immunostimulatory, according to previous studies. These changes will be detailed and discussed in the following sections. The genes upregulated in earlier promastigote differentiation stages may be used to design intra-vector control strategies, and those upregulated at the end of differentiation may be vaccine candidates. These expression patterns are not always mutually exclusive. A given protein may be a vaccine candidate whenever the steady-state levels are sufficient for immunization, even when their levels decrease compared to other growth phases. The LACK antigen is an example. Western blot-validated downregulation in *L. donovani* promastigotes indicates that LACK is upregulated in early logarithmic phase promastigotes. Constant low expression levels remain until the end of differentiation (Fig. 6), as confirmed in *L. infantum* and *C. fasciculata*. Therefore, these data support that LACK plays determinant roles in all promastigote differentiation stages and may be used in intra-vector control, not only as a vaccine. The gene expression criterion is effective to select vaccine candidates only when combined with other criteria, such as immunogenic properties and biological role (Alcolea et al. 2016a). LACK elicits a Th1 response (Ramiro et al. 2003; Ramos et al. 2008), and relatively low expression levels in differentiated promastigotes are sufficient to protect against infection.

### Overview of the differentially abundant proteins: GO enrichment analysis

**Fig. 7:**
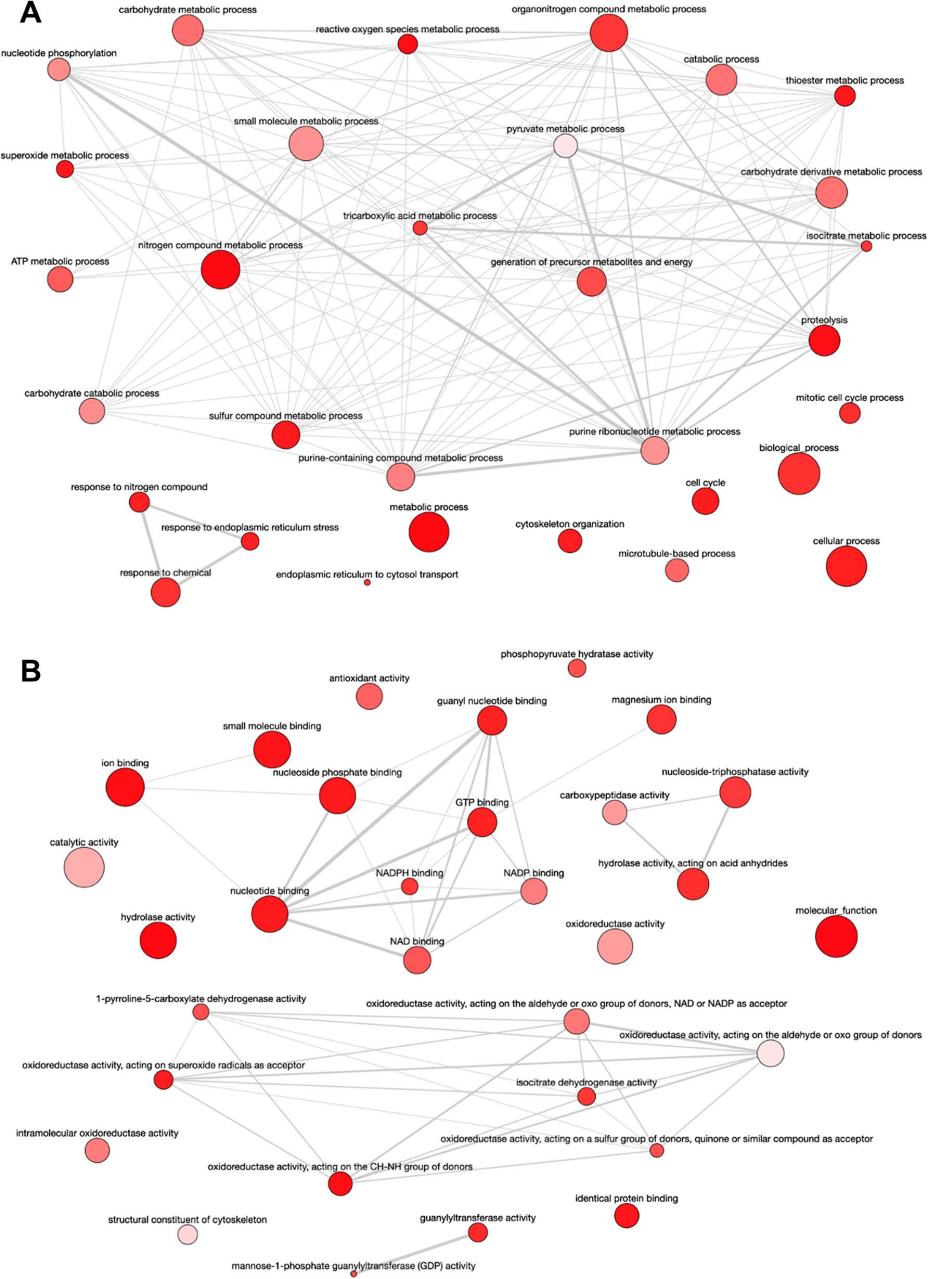
Overview of the differentially abundant proteins: GO enrichment analysis. **A** Mid-logarithmic phase promastigotes increase the levels of a set of proteins enriched in GOBP terms involved in mitosis, cytoskeleton organization and microtubule-based processes, proteolysis, carbohydrate catabolic processes, the tricarboxylic acid cycle (TCA), energy obtention, ATP metabolism, generation of precursor metabolites, nucleotide metabolism, metabolism of nitrogen and sulfur compounds, response to nitrogen compounds, reactive oxygen species (ROS) metabolic processes, superoxide metabolism, and transport from the endoplasmic reticulum to the cytosol. **B** GOBP terms are associated with GOMF terms reflecting the same types of processes easily recognizable in the interactive graph

Compared to early logarithmic phase promastigotes, mid-logarithmic phase promastigotes increase the levels of a set of proteins enriched in GOBP terms involved in mitosis, cytoskeleton organization and microtubule-based processes, proteolysis, carbohydrate catabolic processes, the tricarboxylic acid cycle (TCA), energy obtention, ATP metabolism, generation of precursor metabolites, nucleotide metabolism, metabolism of nitrogen and sulfur compounds, response to nitrogen compounds, reactive oxygen species (ROS) metabolic processes, superoxide metabolism, and transport from the endoplasmic reticulum to the cytosol (Fig. 7A; SI: Fig. 12A). These GOBP terms are associated with GOMF terms reflecting the same types of processes easily recognizable in the interactive graph shown in Fig. 7B and SI: Fig. 12B.

**Fig. 8:**
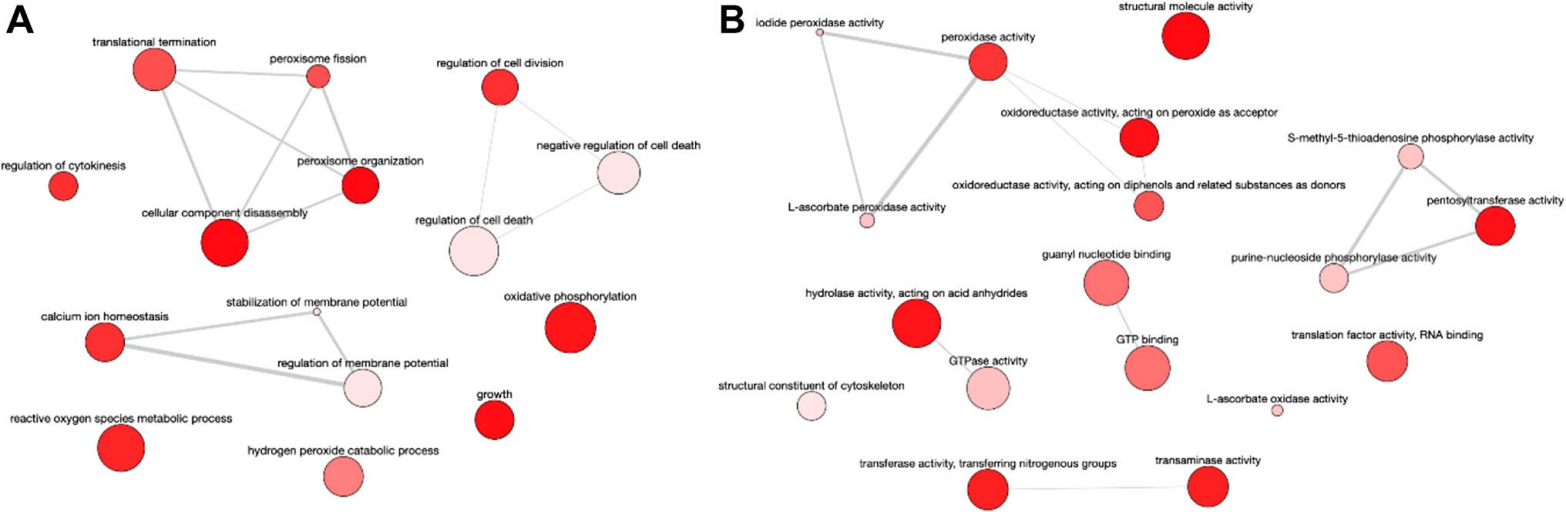
**A** GOBP terms associated with the set of proteins that decrease in mid-logarithmic phase promastigotes compared to the early logarithmic phase are involved in translational termination, regulation of cytokinesis, peroxisome fission, regulation of cell division and cell death, stabilization of membrane potential, calcium ion homeostasis, reactive oxygen species metabolic process, hydrogen peroxide catabolic process, growth, and oxidative phosphorylation. **B** Corresponding GOMF terms are structural molecule activity, L-ascorbate peroxidase activity, oxidoreductase activity acting on peroxide as acceptor and on diphenols and other substances as donors, guanylyl nucleotide-binding, GTP-binding, hydrolase activity, GTPase activity, transaminase activity, translation factor activity, cytoskeleton, and S-methyl-5-thioadenosine phosphorylase activity

The GOBP terms associated with the set of proteins that decrease in mid-logarithmic phase promastigotes compared to the early logarithmic phase are involved in translational termination, regulation of cytokinesis, peroxisome fission, regulation of cell division and cell death, stabilization of membrane potential, calcium ion homeostasis, reactive oxygen species metabolic process, hydrogen peroxide catabolic process, growth, and oxidative phosphorylation (Fig. 8A; SI: Fig. 13A). The corresponding GOMF terms are structural molecule activity, L-ascorbate peroxidase activity, oxidoreductase activity acting on peroxide as acceptor and on diphenols and other substances as donors, guanylyl nucleotide-binding, GTP-binding, hydrolase activity, GTPase activity, transaminase activity, translation factor activity, cytoskeleton, and S-methyl-5-thioadenosine phosphorylase activity (Fig. 8B; SI: Fig. 13B).

**Fig. 9:**
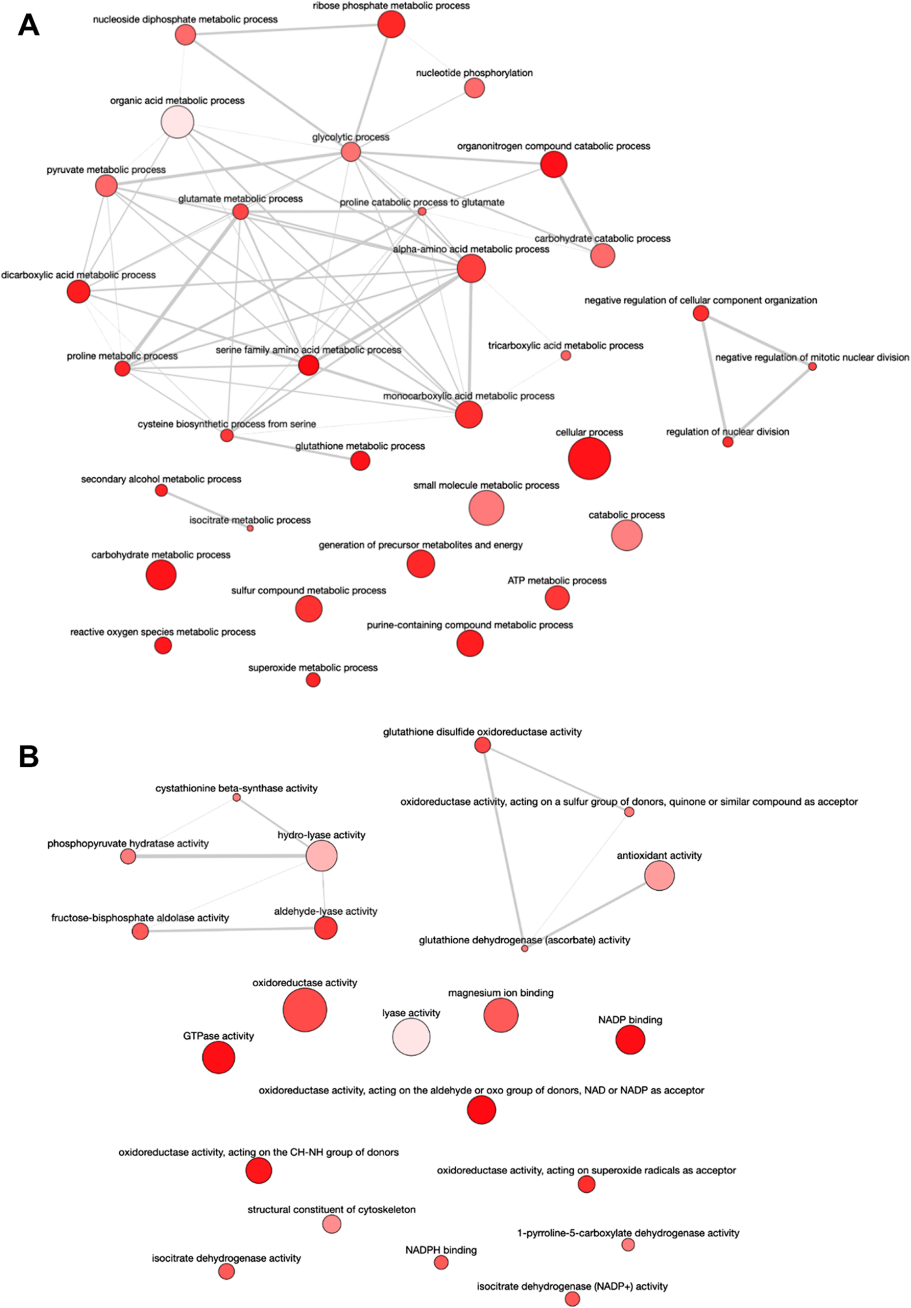
**A** The GOBP terms increased in stationary phase promastigotes are related to carbohydrate, amino acid, and nucleotide metabolic processes, catabolism, the TCA, processes related to redox homeostasis, and negative regulation of mitosis. **B** GOMF terms reflect these biological processes

The GOBP terms increased in stationary phase promastigotes are related to carbohydrate, amino acid, and nucleotide metabolic processes, catabolism, the TCA, processes related to redox homeostasis, and negative regulation of mitosis (Fig. 9A; SI: Fig. 14A). The GOMF terms reflect these biological processes through the following activities (Fig. 9B; SI: Fig. 14B): glutathione disulfide oxidoreductase, glutathione dehydrogenase (ascorbate), oxidoreductase on superoxide radicals as acceptor, antioxidant, cystathionine beta-synthase (involved in glutathione biosynthesis), delta-1-pyrroline-5-carboxylate, isocitrate dehydrogenase, GTPase, lyase; fructose-bisphosphate aldolase, phosphopyruvate hydratase, aldehyde-lyase, NADP- and NADPH-binding, and lyase.

**Fig. 10:**
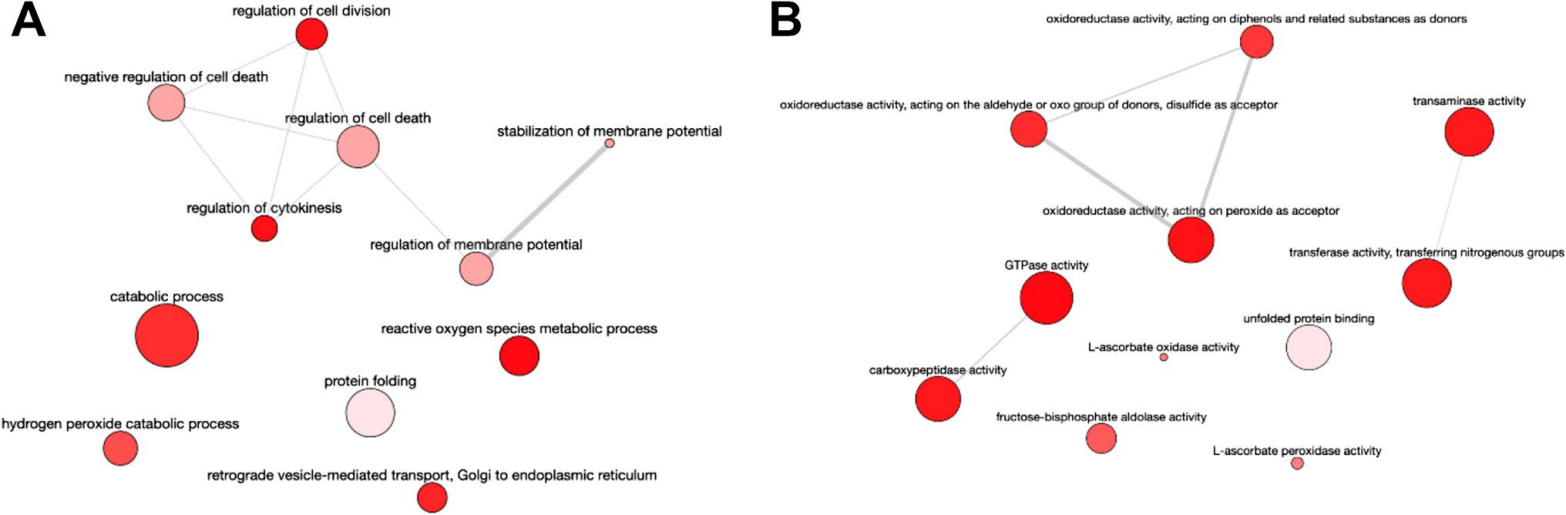
**A** GOBP terms that decrease in stationary phase promastigotes are related to the regulation of cell division and cytokinesis, negative regulation of cell death, stabilization of membrane potential, reactive oxygen species metabolic processes, including hydrogen peroxide, catabolic processes, and retrograde vesicle-mediated transport. **B** GOMF terms in this set are L-ascorbate peroxidase activity, oxidoreductase on peroxide as acceptor, oxidoreductase activity on the aldehyde or oxo group of donors, transaminase activity, carboxypeptidase activity, fructose-bisphosphate aldolase activity (associated to oxidoreductase activity acting on diphenols and related substances as donors), unfolded protein-binding, and GTPase activity

The GOBP terms that decrease in stationary phase promastigotes are related to the regulation of cell division and cytokinesis, negative regulation of cell death, stabilization of membrane potential, reactive oxygen species metabolic processes, including hydrogen peroxide, catabolic processes, and retrograde vesicle-mediated transport (Fig. 10A; SI: Fig. 15A). The associated GOMF terms in this set are L-ascorbate peroxidase activity, oxidoreductase on peroxide as acceptor, oxidoreductase activity on the aldehyde or oxo group of donors, transaminase activity, carboxypeptidase activity, fructose-bisphosphate aldolase activity (associated to oxidoreductase activity acting on diphenols and related substances as donors), unfolded protein-binding, and GTPase activity (Fig. 10B; SI: Fig. 15B).

### Gene expression regulation and protein folding

The glycolytic enzyme enolase is more abundant in the mid-logarithmic (day 4) and stationary (day 6) phase promastigotes (Table 1) than in the reference condition (early logarithmic phase promastigotes, day 2). The parasite's enolase is a versatile protein that participates in transcription, protein folding (i.e., chaperone), cell migration, and plasminogen reception (Fonseca et al. 2014). The differential gene expression data don't provide information about which of these processes is favored by enolase upregulation. Interestingly, enolase is an antigen and probably a virulence factor in *Leishmania* spp. (Fonseca et al. 2014; Avilan et al. 2011; Vanegas et al. 2007; Abanades et al. 2012). The enolase increase in differentiated promastigotes and the role as an antigen suggest that this is a vaccine candidate.

The gene copies LINF_340014000 and LINF_340014200 encode for elongation factor 1β (EF1β), which increases in the early logarithmic phase of *L. donovani* promastigotes (Table 1). Conversely, the levels of EF1β encoded by the gene LINF_360020500 are constitutive in promastigotes of this species (Alcolea et al. 2019b). The LINF_340014000/14200 EF1β differs from LINF_360020500 EF1β in the N-terminal region (~ 100 and ~ 71 first amino acids, respectively; SI: Fig. 16). Elongation factor 2 (EF2) reaches higher levels at the beginning of *L. donovani* promastigote growth and differentiation (Table 1). The EF2 N-terminal domain is an immunostimulatory molecule protecting BALB/c mice in combination with CpG oligodeoxynucleotides as molecular adjuvants (Agallou et al. 2018). This protein stimulates the protective Th1 response against the parasite. (Gupta et al. 2007; Kushawaha et al. 2011). The EF2 levels decrease in mid-logarithmic and stationary phase promastigotes, and EF2 is present in infective promastigotes, in which this molecule may be a target in an immunized animal in the early infection steps.

The endoribonuclease L-PSP contributes to parasite survival (Fonseca et al. 2014). A mammalian ortholog is a potent protein synthesis inhibitor (Morishita et al. 1999). This activity may be related to metacyclogenesis (mid-logarithmic phase), as EndoL-PSP upregulation suggests, because differentiation probably involves translational efficiency modulation, as confirmed in *Trypanosoma* spp. (Jensen et al. 2014; Smircich et al. 2015). Therefore, the endoribonuclease L-PSP may contribute to survival affecting differentiation through gene expression-mediated modulation.

The levels of several chaperones change across the growth and differentiation of *L. donovani* promastigotes (Table 1). The heat shock protein 110 (HSP110) levels are higher on day 2. All other mentioned HSPs increase in later stages. Chromosome 28 encodes an HSP70 gene tandem array composed of two copies (spots LdK.2905, LdK.3901, and LdK.4902). HSP70 increases in mid-logarithmic and stationary phase promastigotes. These findings suggest that this protein from the HSP70 family plays a determinant role during the intermediate stages of *L. donovani* promastigote differentiation. HSP70 proteins are also relevant for parasite survival (Fonseca et al. 2014). Also, three hsp70-related mitochondrial precursor paralogs increase during the ongoing growth and differentiation, one on day 4 and two on day 6. The HSP83-17 protein also increases on day 6. HSP70 and HSP83 are immunostimulatory proteins triggering specific B cell proliferation (Echeverria et al. 2001; Rico et al. 1999, 1998, 2002).

The T-complex protein 1 (TCP1) *γ*, *δ*, and *η* subunits decrease in the stationary phase of *L. donovani* promastigotes. The same profile was observed for TCP1γ protein levels in *L. infantum* (Alcolea et al. 2011). TCP1 is a ring-shaped chaperonin complex responsible for folding certain nascent or incorrectly folded proteins such as tubulins in an ATP-dependent manner. This complex is active in *L. donovani* because refolding of unfolded luciferase was achieved using a homo-oligomeric LdTCP1γ complex alone without the LdTCP1 partner units. LdTCP1γ also interacts with tubulins (Bhaskar et al. 2015).

The protein disulfide isomerase 2 (PDI2) is an enzyme facilitating correct protein folding in the ER lumen. PDI catalyzes disulfide bond reduction and formation, protein isomerization, and protein folding. Bacitracin inhibits isomerase and reductase activity, which leads to promastigote and amastigote growth. Other compounds inhibit PDI activity in *L. major*. Therefore, PDI2 is a potential drug target (Ben Khalaf et al. 2012). Mid-logarithmic phase *L. donovani* promastigotes upregulate PDI2 (Table 1). An intense metabolic activity leading to protein synthesis is a feature of this growth phase.

### Cell division and protein degradation

The transitional ER ATPase (TER-ATPase; CDC48 in *Saccharomyces cerevisiae*) is involved in the segregation of macromolecular complexes, such as chromatin, membranes, and protein assemblies (e.g., proteasome) (Druck et al. 1995; Rabouille et al. 1995). Therefore, the high relative expression levels of TER-ATPase found in actively growing mid-logarithmic phase *L. donovani* promastigotes (Table 1) match with a role in cell cycle progression. The proteasome α7 subunit is upregulated in early logarithmic phase promastigotes, and two carboxypeptidases increase in the mid-logarithmic phase.

The replication factor A 51 KDa subunit (RPA^51^) levels are null in the stationary phase. RPA^51^ is part of a heterotrimeric complex involved in single-stranded DNA intermediate stabilization in the DNA replication process during the cell cycle S phase and damaged DNA repair. This information is consistent with RPA^51^ gene downregulation in the stationary phase, where the population replication rate is null.

### Metabolism and glycoconjugate biosynthesis

The glycolytic enzymes fructose-1,6-bisphosphate aldolase (ALD), triosephosphate isomerase (TPI), cytosolic glyceraldehyde-3-phosphate dehydrogenase (cGAPDH), and enolase are upregulated in advanced differentiation stages. Specifically, TPI, cGAPDH, and enolase increase in the mid-logarithmic and stationary phase, whereas ALD only increases in the stationary phase. Enolase is involved in a compendium of biological roles, contributing to transcription, protein folding (i.e., chaperone), cell migration, and plasminogen reception (Fonseca et al. 2014), which facilitates parasite survival. This protein is an antigen, a virulence factor, and a probable vaccine candidate.

The pyruvate dehydrogenase complex E1β subunit (PDC-E1β) is upregulated in mid-logarithmic phase *L. donovani* promastigotes. On the contrary, the dihydrolipoamide acetyltransferase (DHLAT) PDC component is upregulated in the early and mid-logarithmic phases (Fig. 5, Table 1). The isocitrate dehydrogenase (ICDH) increases in the mid-logarithmic and stationary phases. The respiratory chain component iron-sulfur cluster 2Fe-2S protein is upregulated in the early logarithmic phase of promastigotes. On the grounds of observations in pathogenic *E. coli* strains and *Mycobacterium tuberculosis* (Brandes et al. 2007; Rhee et al. 2005), as well as proven resistance of amastigotes and promastigotes to NO, we proposed a hypothesis relating differential abundance of glycolytic, PDC, Krebs cycle, and respiratory chain proteins with resistance to NO in *L. amazonensis* (Alcolea et al. 2016b), a species that causes American Cutaneous Leishmaniasis (ACL).

The GDP-mannose pyrophosphorylase (GDP-MP) is upregulated in the mid-logarithmic phase. This enzyme is crucial for LPG biosynthesis, glycosyl inositol phospholipids (GIPLs), and other surface glycoconjugates. The *L. mexicana* promastigote GDP-MP is essential for virulence (Davis et al. 2004). GDP-MP downregulation in the stationary phase is consistent with these observations because LPG biosynthesis is only required before the differentiation process concludes.

Methylthioadenosine phosphorylase (MTAP) is an enzyme involved in purine and polyamine salvage biosynthesis pathways (Bacchi et al. 1991). This enzyme is important in trypanosomatids because these parasites lack the *de novo* purine and polyamine biosynthesis pathways (Kouni 2003) and require spermidine for trypanothione biosynthesis. These differences with the mammalian hosts are important and suggest that MTAP is a potential therapeutic target (Datta et al. 2008; Singh et al. 2012). 5′-methylthioadenosine (MTA) is the natural MTAP substrate, which is cleaved to 5-methylthioribose-1-phosphate (MTRP) and adenine (Bacchi et al. 1991; Bertino et al. 2011). The MTAP gene is strongly upregulated at the protein level in *L. donovani* intracellular amastigotes (Pescher et al. 2011). MTAP might be a drug target according to this expression profile, the mentioned differences between trypanosomatids and mammalian hosts in purine, polyamine, and methionine biosynthesis, and high druggability indexes (Abid et al. 2017). The MTAP expression peak occurs in the early logarithmic phase of *L. donovani* promastigotes. Therefore, this protein may also be a target for intra-vector parasite control.

The cystathionine β-synthase (CβS) catalyzes the first step of the two-reaction reverse trans-sulfurylation cysteine biosynthesis pathway. CβS substrates are homocysteine and serine, and cysteine γ-lyase converts the product into cysteine. The CβS is more abundant in the stationary phase (Table 1) and participates in resistance to oxidative stress (Romero et al. 2015). Therefore, this enzyme is part of the protein repertoire increased in differentiated promastigotes.

The aspartate aminotransferase (ASAT) from *Leishmania* spp. is a broad-specificity enzyme because L-aspartate, L-tyrosine, L-alanine, and L-tryptophan are substrates. The optimal temperature for ASAT activity is 29.5ºC in *Leishmania* (Fair and Krassner 1971), close to the promastigote growth optimum (26–27 °C). This enzyme is more abundant in the early logarithmic phase (Table 1). In this phase, amino acid demand for protein biosynthesis is higher. The δ-pyrroline-5-carboxylate dehydrogenase is an enzyme involved in proline catabolism increased in mid-logarithmic and stationary phase promastigotes (Table 1). The transcript encoding for this enzyme also increases in highly infective promastigotes isolated from the sand fly stomodeal valve (Alcolea et al. 2016c). Therefore, this enzyme increases in the ongoing promastigote differentiation.

### Cytoskeleton

The levels of several α- and β-tubulins increase during promastigote growth and differentiation, whereas other variants decrease. These expression profiles may be related to body length decrease and flagellar length increase at this stage. The flagellar radial spoke protein reaches its maximum levels in mid-logarithmic phase promastigotes, where more elongated parasites called nectomonads are predominant (Lei et al. 2010). Fully differentiated promastigotes are shorter but show a higher cell body:flagellum length ratio. The paralyzed flagellar protein 16 increases in mid-logarithmic phase promastigotes.

### Redox homeostasis

The *L. donovani* LINF_340014000/14200 EF1β (Table 1) increases in early logarithmic phase promastigotes (Table 1), as well as the *L. pifanoi* ortholog (Alcolea et al. 2016d). The LINF_360020500 EF1β paralog is constitutively expressed in *L. donovani* (Alcolea et al. 2019b). On the contrary, the ortholog from the closely related non-pathogenic trypanosomatid *Crithidia fasciculata* is increased in early logarithmic phase (Alcolea et al. 2014). The LINF_340014000/14200 EF1β differs from the LINF_360020500 EF1β in the N-terminal region (~ 100 and ~ 71 first amino acids; SI: Fig. 16), whereas their C-terminal regions are identical and bear the elongation factor 1β function (SSF54984, PF00736, IPR014038, IPR036019, PTHR11595). The N-terminal domain lacks annotated domains in both cases. Hence, this region may be responsible for the trypanothione S-transferase activity reported by Vickers et al. (Vickers and Fairlamb 2004; Vickers et al. 2004), and cadmium-induced stress increases LINF_360020500 EF1β transcript levels. The LINF_340014000/14200 EF1β trypanothione S-transferase activity is represented in Fig. 5 as EF1β* to distinguish this protein from the constitutive LINF_360020500 EF1β in *L. donovani*.

Three almost identical genes encode for tryparedoxin peroxidases (TrxPs) according to multiple alignments (Alcolea et al. 2019b). These enzymes can reduce reactive oxygen species (ROS) in the presence of reduced tryparedoxin (TXN[SH_2_]), which is oxidized (TXN[S]) during this reaction. TXN[SH_2_] is regenerated by reduced trypanothione (T[SH_2_]), which is recycled by the NADPH-dependent enzyme trypanothione reductase (TryR) (Day 2009; Flohe et al. 1999; Gretes et al. 2012). A TXN gene was also confirmed to be constitutively expressed in promastigotes (Alcolea et al. 2019b), whereas a different TXN called AhpC/TSA family thioredoxin-like protein is upregulated in early logarithmic phase promastigotes. Constitutive levels of the iron superoxide dismutase (Fe-SOD) paralogs (SI: Fig. 17) were also found (Alcolea et al. 2019b), except for LINF_320033200. The present study has revealed that the LINF_320033200 Fe-SOD paralog (Fe-SOD*) increases in mid-logarithmic and stationary phase promastigotes (Table 1). The Fe-SOD enzymes are functionally coupled to TrxPs through superoxide anion reduction (Fig. 5) generated in reactions catalyzed by enzymes like the ribonucleotide reductase (RNR). In the non-pathogenic trypanosomatid *Crithidia fasciculata*, Fe-SOD and TrxP are also upregulated (Alcolea et al. 2014).

Ascorbate peroxidase (APX) is a heme-dependent enzyme initially found in cyanobacteria, plants, and algae which catalyzes L-ascorbate oxidation to dehydroascorbate using hydrogen peroxide as electron donor. This enzyme participates in the ascorbate–glutathione cycle in plants, which is also present in trypanosomatids and allows for hydrogen peroxide elimination (Caverzan et al. 2012). Functional APX-like enzymes have been found in trypanosomatids. For example, a *Trypanosoma cruzi* APX (TcAPX) can protect the parasite against hydrogen peroxide-induced stress, which was demonstrated by comparing benznidazole sensitive (BZS) and resistant (BZR) strains. APX levels increase in BZR compared to BZS strains (Nogueira et al. 2012). TcAPX catalyzes the electron transfer from reduced ascorbate. NADPH oxidation via trypanothione-TryR recycles this molecule (Fig. 5) (Wilkinson et al. 2002). TcAPX is a type A heme-dependent hybrid with the cytochrome c peroxidase (TcAPX-CCP), using ferrocytochrome c and ascorbate as electron donors (Hugo et al. 2017). In *T. cruzi*, TcAPX-CCP is located in the amastigote ER (Wilkinson et al. 2002), mitochondrion, and nucleus (Hugo et al. 2017). The genus *Leishmania* also contains this enzyme. A potential antioxidant role–scavenging hydrogen peroxide and superoxide anion has been found in *L. major*. LmAPX-CCP activity using ferrocytochrome c is considerably higher than using ascorbate as the electron donor, suggesting a role similar to yeast CCP, which consists of hydrogen peroxide and superoxide anion detoxification in the mitochondrion (Adak and Pal 2013). Mitochondria contribution to ROS production in non-photosynthetic organisms is much higher than in plants. The LdAPX peak level is found in the early logarithmic phase (Fig. 5, Table 1), suggesting that intense metabolic activity at the beginning of the growth curve requires hydrogen peroxide removal. TryR is not the only enzyme capable of oxidizing NADPH for the LdAPX reaction in trypanosomatids. The thiol-dependent reductase 1 (TDR1) can reduce ascorbate by transferring reducing power from NADPH. The TDR1 level increase has been observed in *L. donovani* mid-logarithmic and stationary phase promastigotes, whereas the TryR levels remain constitutive in all growth phases, like in *L. amazonensis* (Alcolea et al. 2016b). In summary, these data support that the redox homeostasis proteins changing their levels are APX, AhpC-TXN, EF1β*, two TDR1, and Fe-SOD*, whereas all TrxP and all other TXN and Fe-SOD are constitutively expressed throughout promastigote growth and differentiation. The functional connections between APX, AhpC-TXN, EF1β*, TDR1, and Fe-SOD are represented in Fig. 5.

## Conclusions

This study has revealed the differential abundance of 75 proteins during growth and differentiation. According to previous studies, some are involved in parasite survival (APX, CβS, EF1β*, EF2, endoribonuclease L-PSP, Fe-SOD*, GDP-MP, HSP70, HSP83-17, mHSP70-rel, HSP110, MTAP, TDR1, TER-ATPase, and AhpC-TXN), or are immunostimulatory against *Leishmania* spp. (HSP70, HSP83-17, mHSP70-rel, HSP110, enolase, and LACK) or pathogenic bacteria (ALD, DHLAT, ICDH, PDC-E1α, PDC-E1β, and TPI). The proteins increased at earlier promastigote differentiation stages may be used to design intra-vector control strategies (APX, AhpC-TXN, EF1β*, EF2, HSP110, MTAP, ALD, LACK, DHLAT, and PDC-E1α), and those upregulated at the end of differentiation may be vaccine candidates (CβS, Fe-SOD, endoribonuclease L-PSP, GDP-MP,HSP70, HSP83-17, mHSP70-rel, TDR1, TER-ATPase, enolase, ICDH, PDC-E1β, and TPI). Both groups are not mutually exclusive, and case-by-case characterization is required. For example, although the LACK antigen decreases at the stationary phase, the protein levels may be sufficient to protect against *L. donovani*. We have generated a vaccine against canine leishmaniasis based on the LACK gene. In this study, the *L. donovani* LACK abundance profile found by 2DE-MS/MS has been validated with four independent Western blot replicate experiments.

## Supplementary Information

Below is the link to the electronic supplementary material.

Supplementary file1 (DOCX 4.66 MB)
