# Supplementary material for: An insight into differential protein abundance throughout Leishmania donovani promastigote growth and differentiation
Source: Int Microbiol. 2022 Aug 5;26(1):25–42. doi: 10.1007/s10123-022-00259-4 (PMC9362617; doi:10.1007/s10123-022-00259-4)
Supplement: Supplementary file 1 — Supplementary file1 (DOCX 4.66 MB) [file 10123_2022_259_MOESM1_ESM.docx]

**SUPPLEMENTARY INFORMATION**

**Figure 11. Additional LACK Western blot replicates. The LACK levels decrease during *L. donovani* promastigote growth and differentiation.** Detection and analysis of LACK relative abundance by Western blot in 10 μg total protein extracts using 1:1,000-diluted rabbit anti-LACK polyclonal antibody. The reference protein is gGAPDH, which was detected with a specific monoclonal antibody at a 1:10,000 dilution.

**A**

**
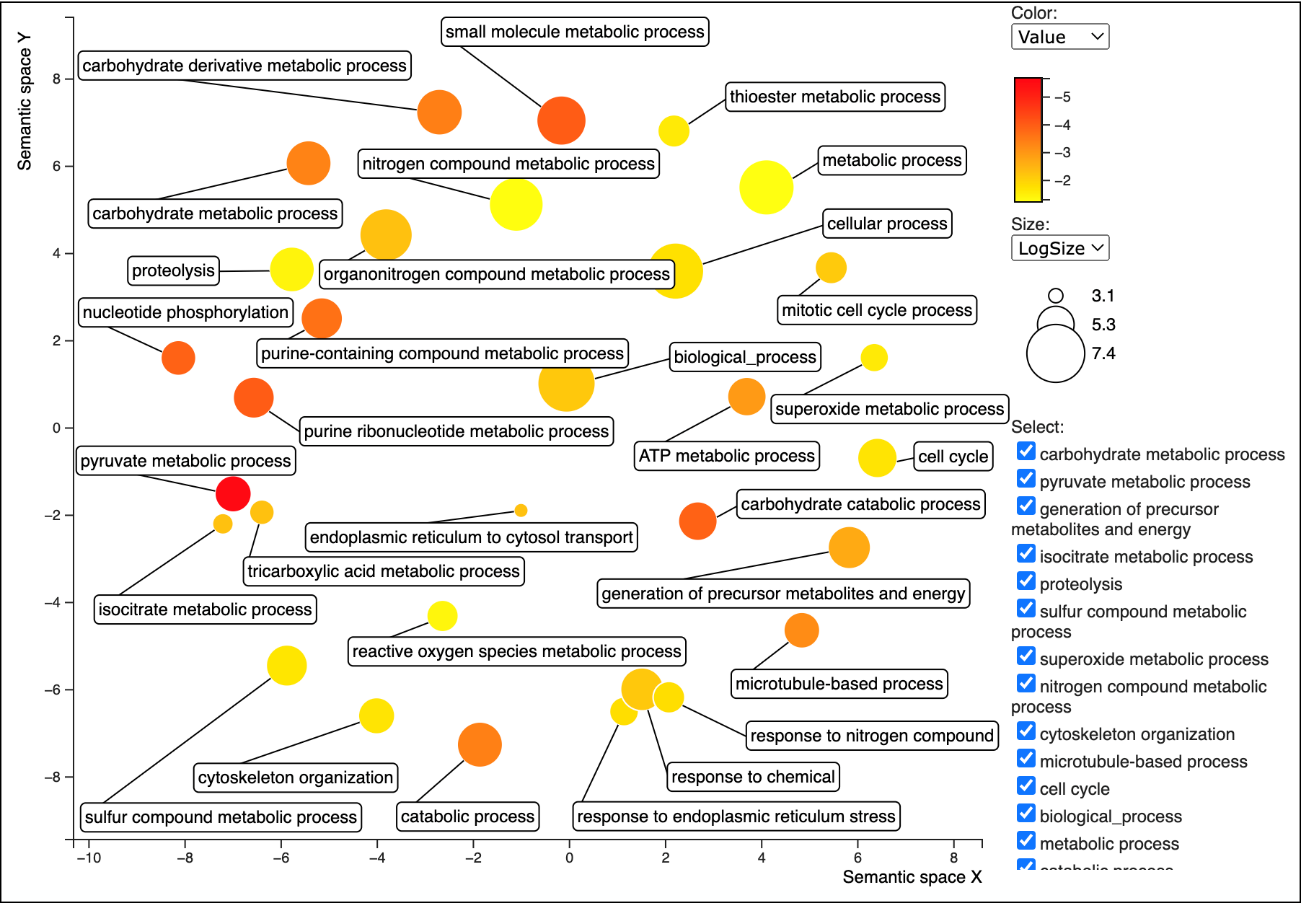
**

**B**


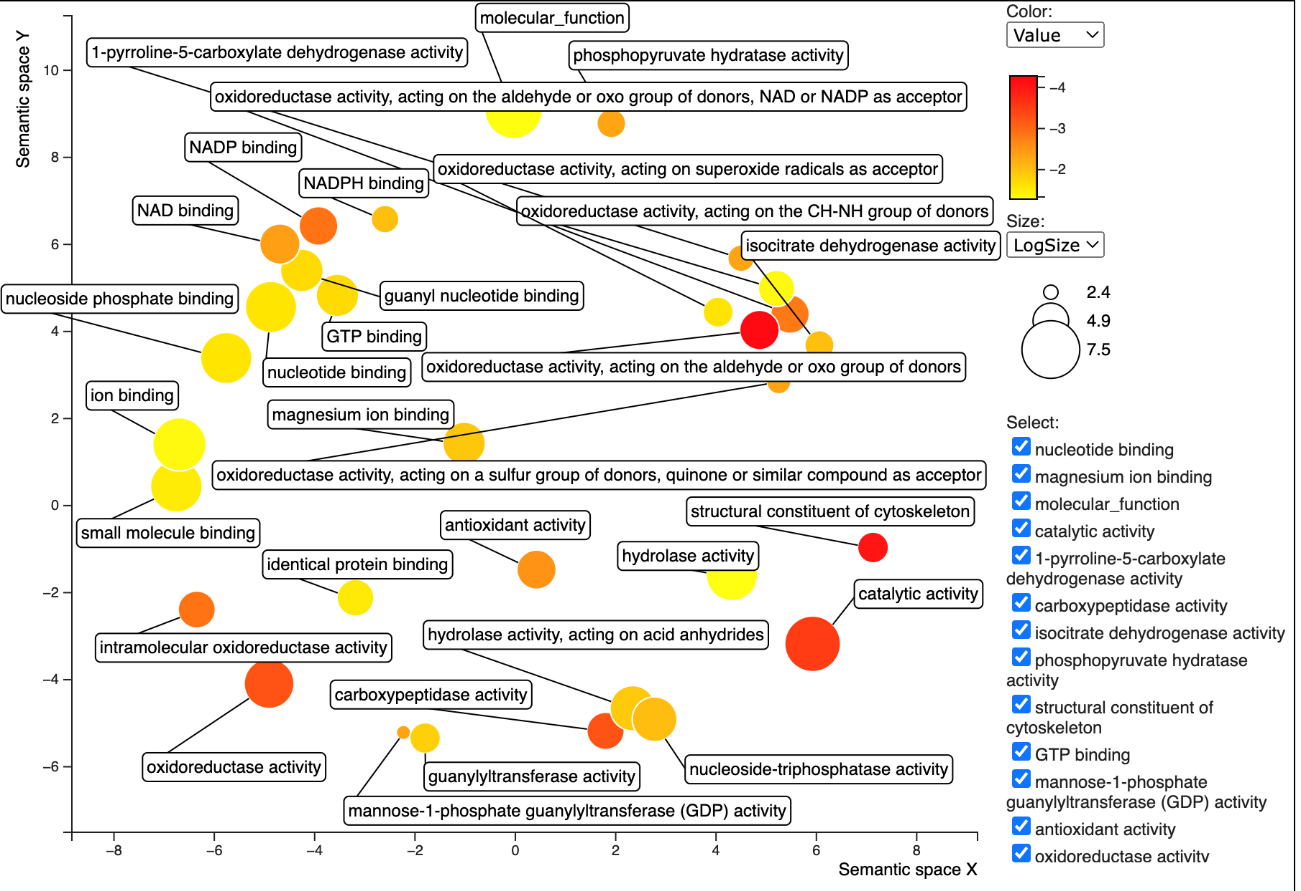


**Figure 12.** **GO term enrichment scatter plot of increased proteins on day 4**. A. GOBP. B. GOMF.

**A**


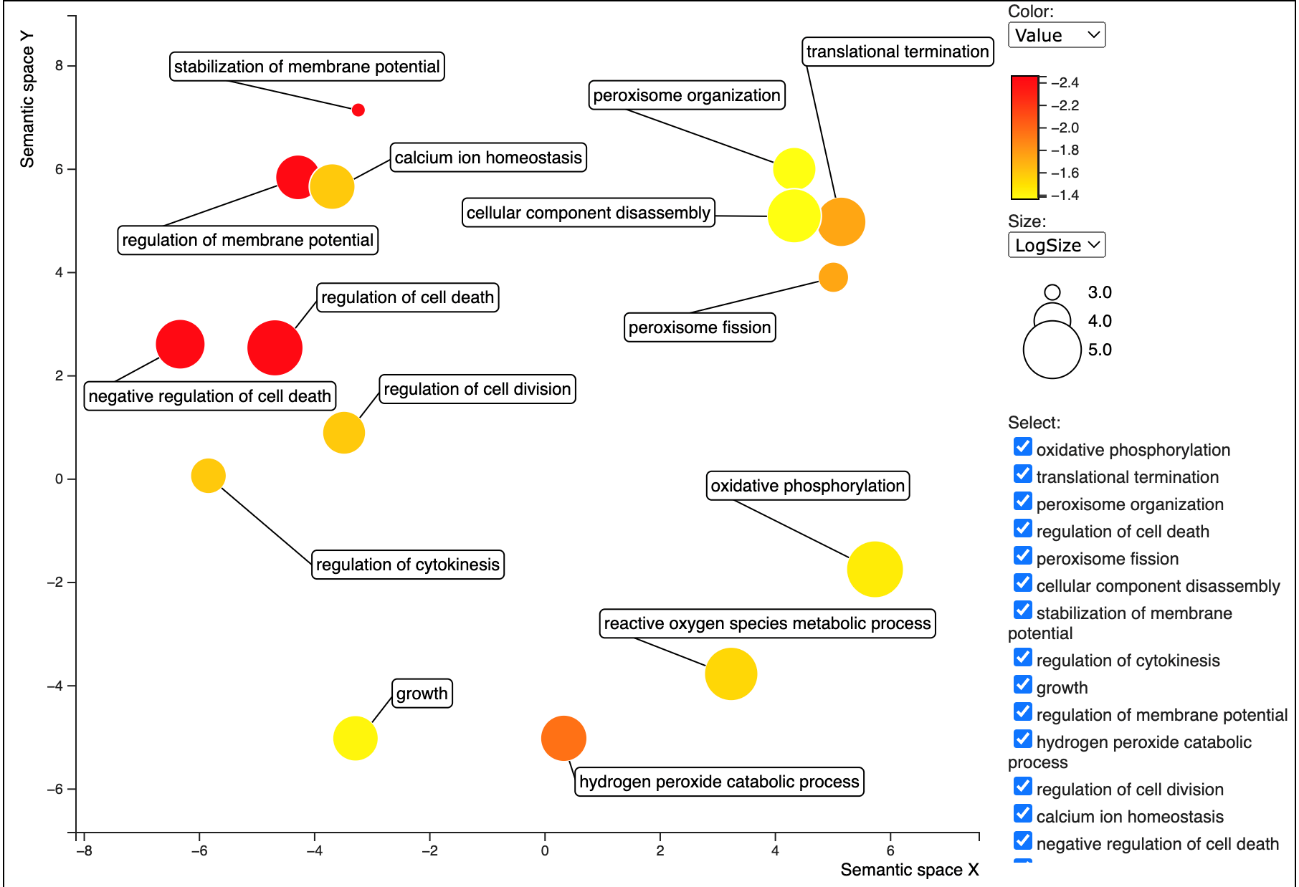


**B**


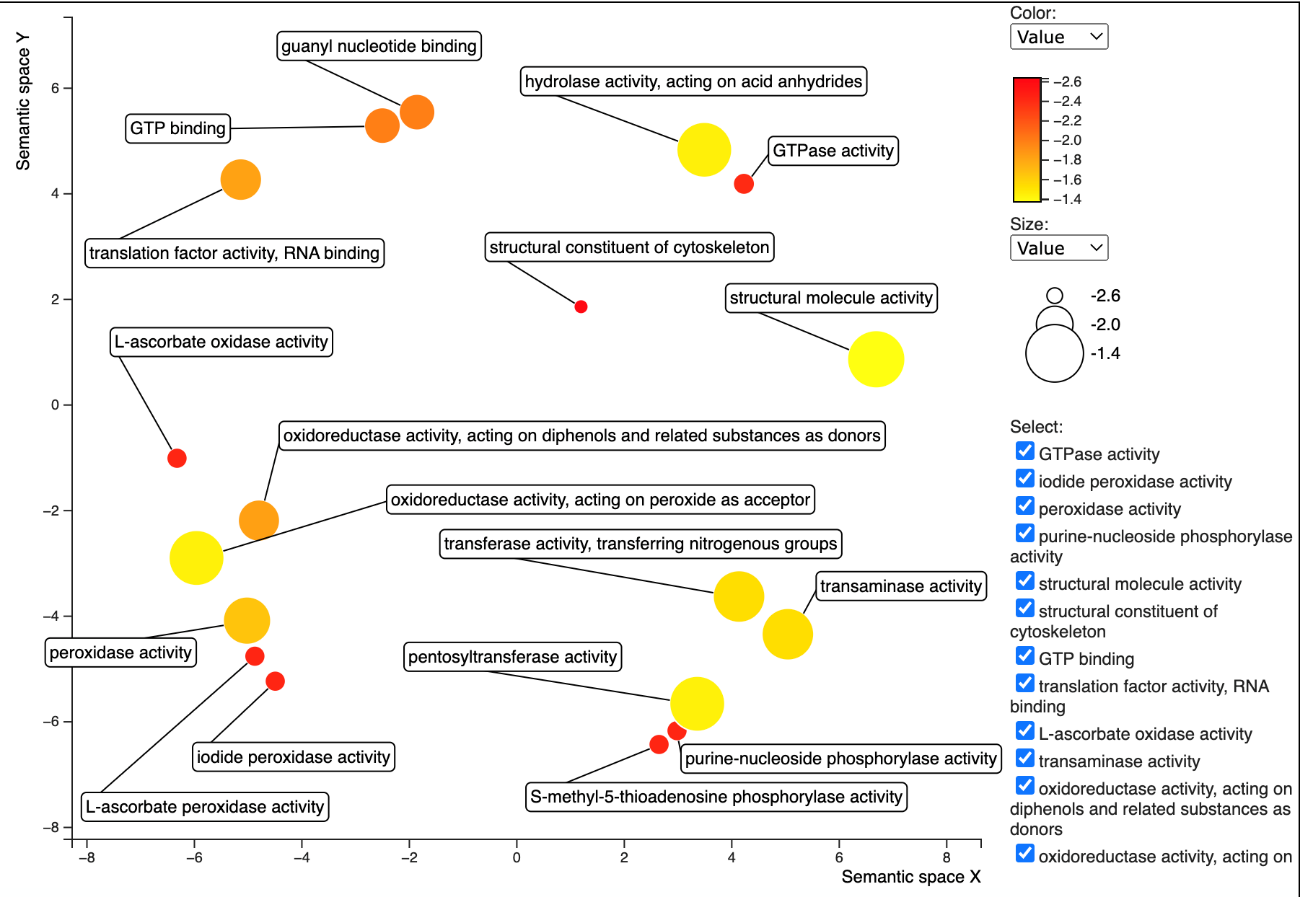


**Figure 13.** **GO term enrichment scatter plot of decreased proteins on day 4**. A. GOBP. B. GOMF.

**A**

**
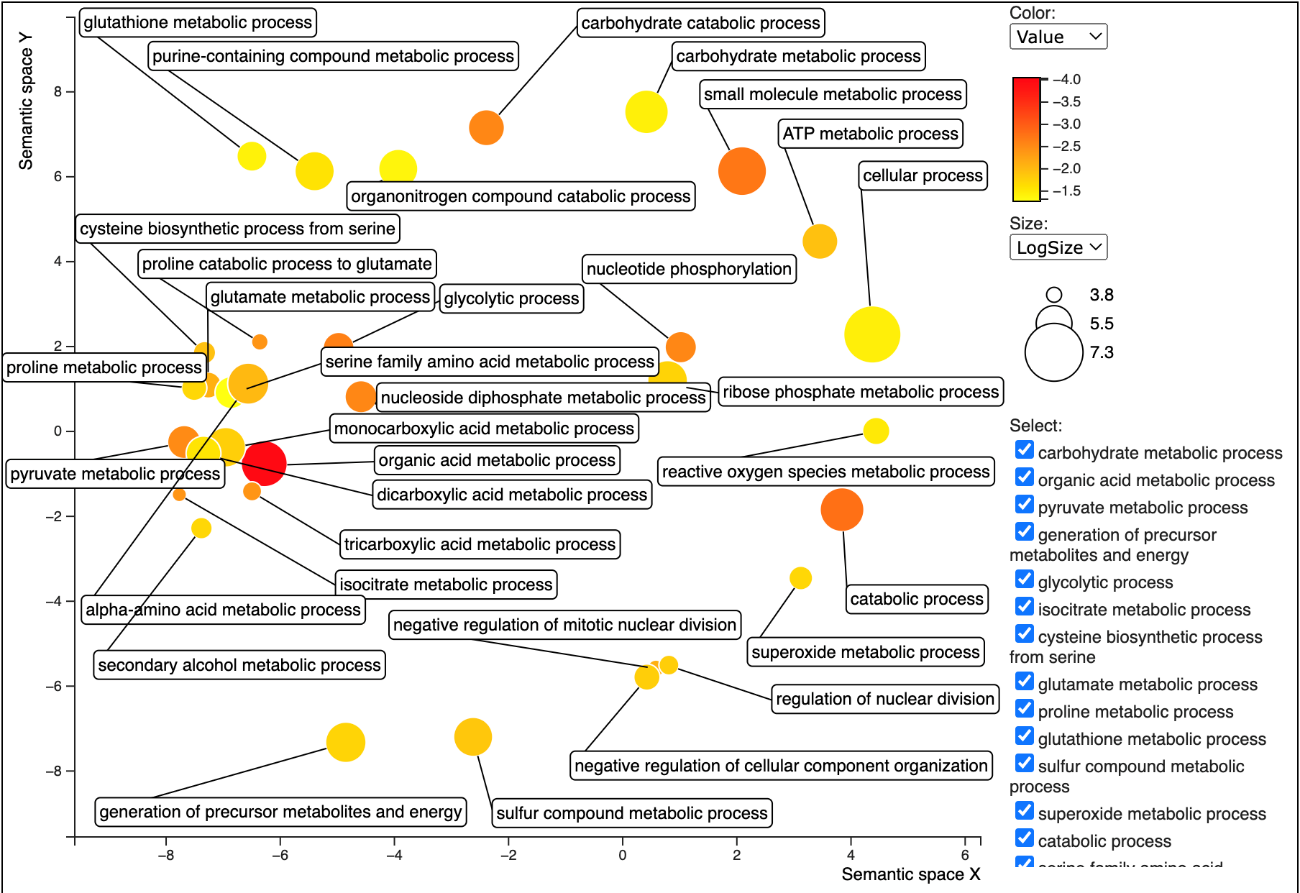
**

**B**

**
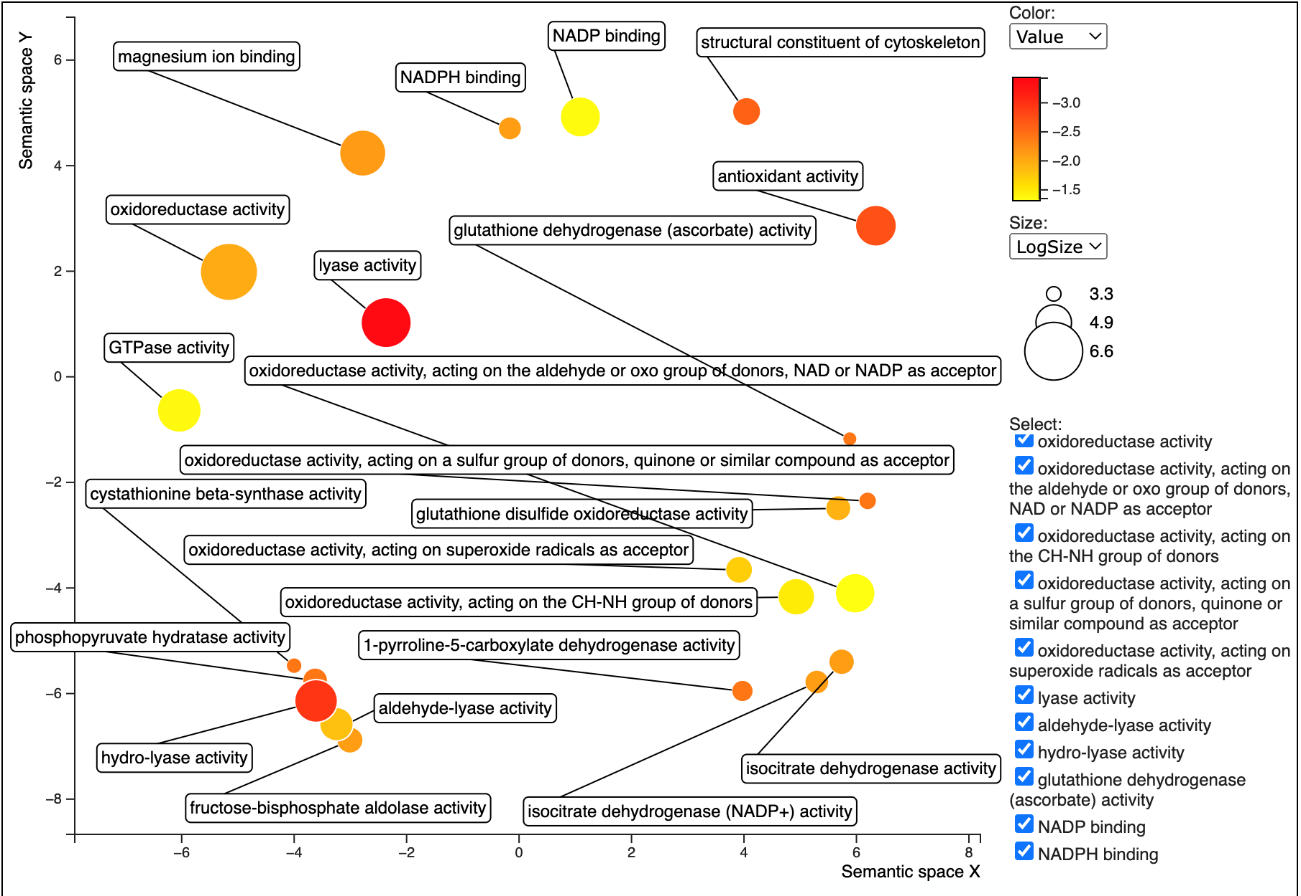
**

**Figure 14.** **GO term enrichment scatter plot of increased proteins on day 6**. A. GOBP. B. GOMF.

**
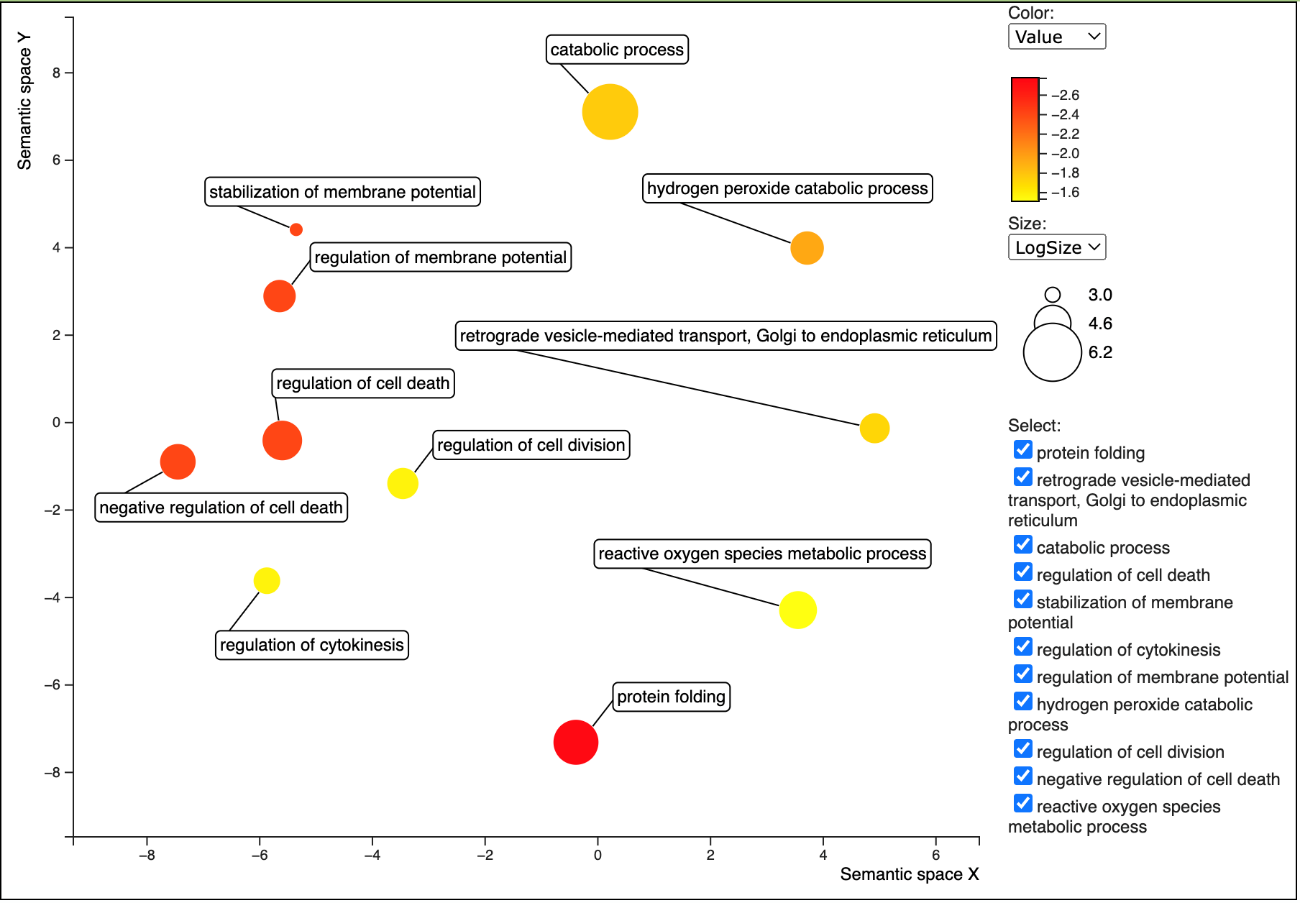
**

**A**

**B**

**
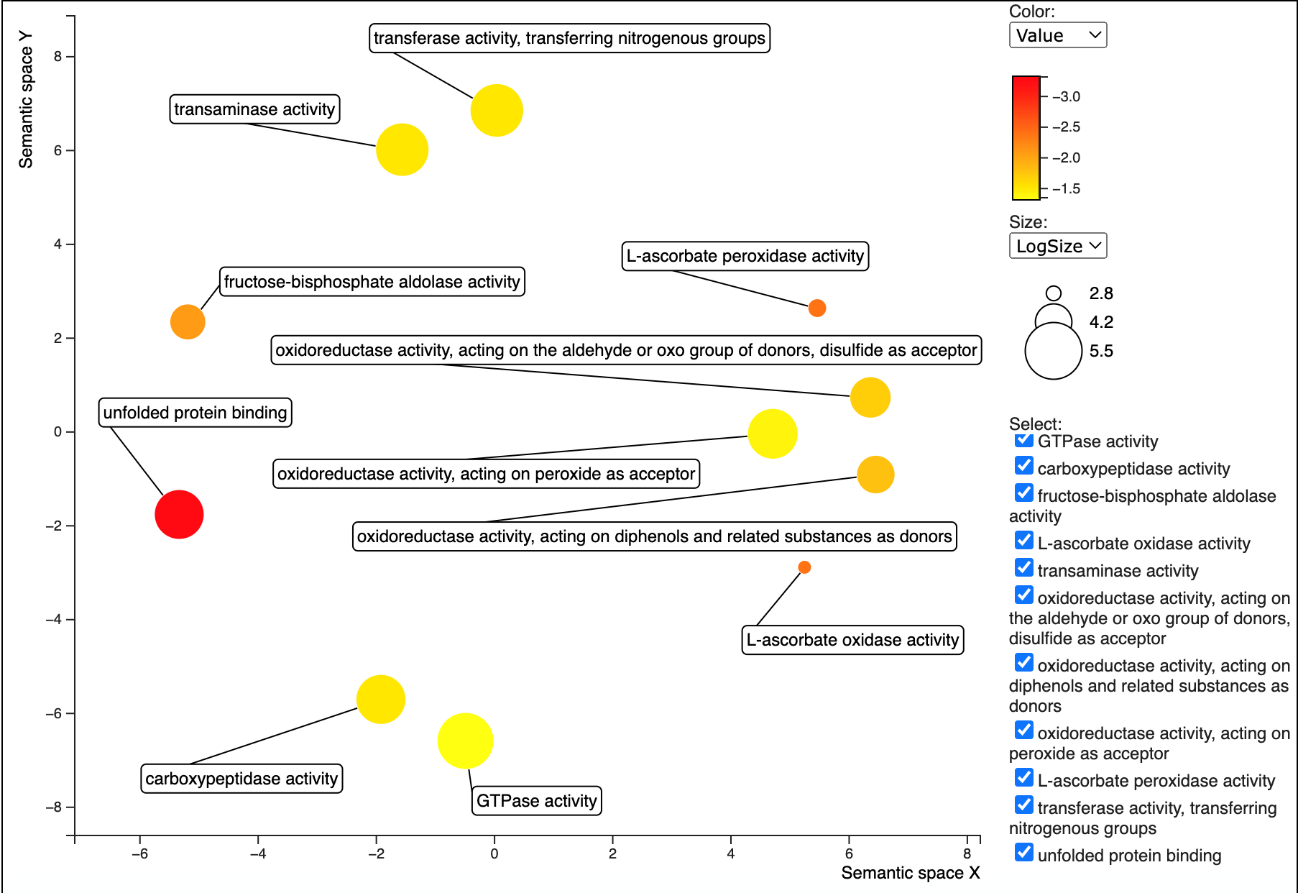
**

**Figure 15.** **GO term enrichment scatter plot of decreased proteins on day 6**. A. GOBP. B. GOMF.


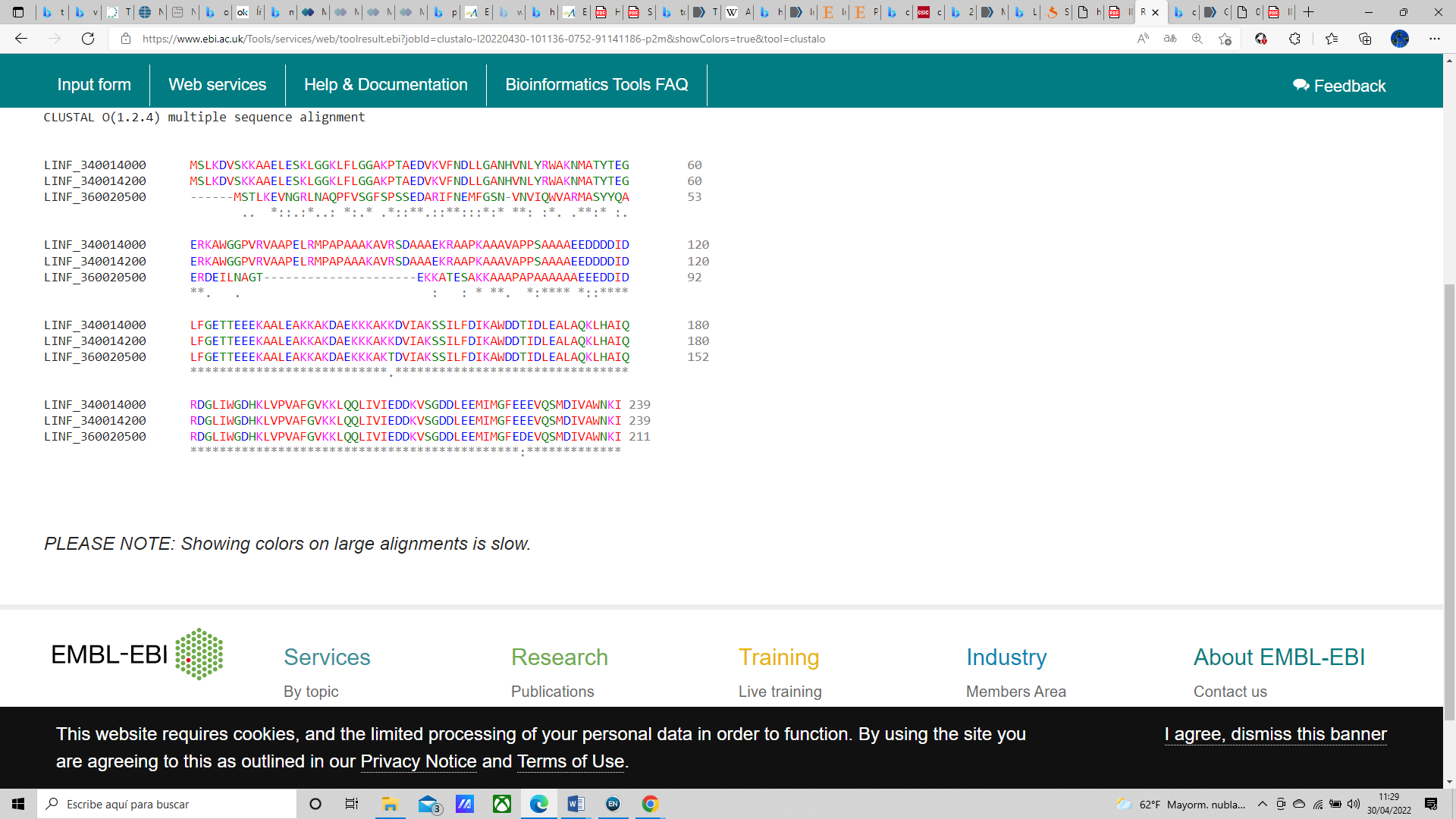


**Figure 16.** **Alignment of the amino acid sequences of the *Leishmania infantum* elongation factor 1β paralogs**. Asterisks indicate identities between all sequences. Semicolons mean strong similarity (>0.5 in the Gonnet PAM 250 matrix) and dots weak similarity (<0.5).


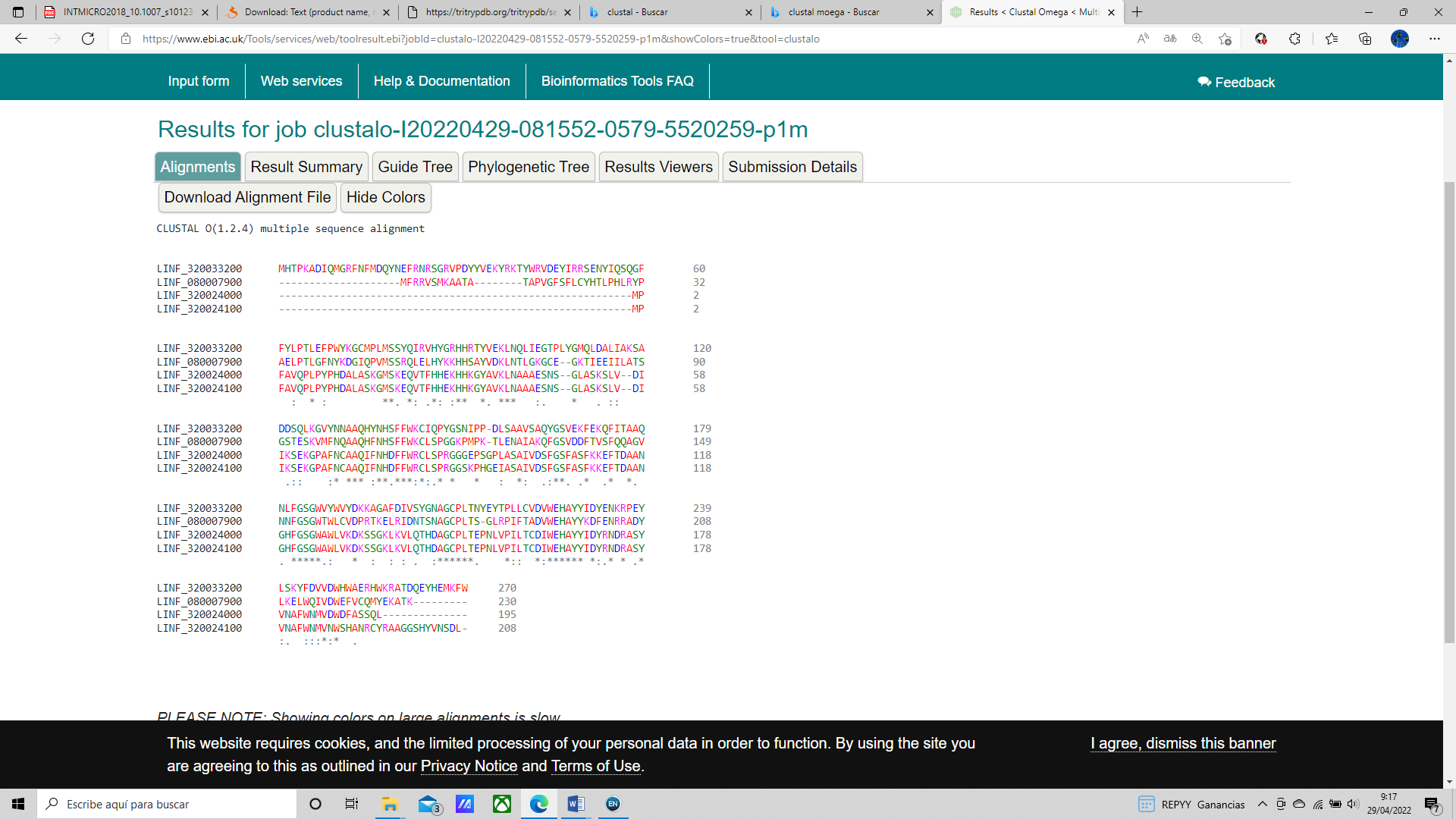


**Figure 17.** **Alignment of the *Leishmania infantum* iron superoxide dismutase amino acid sequences**. Asterisks indicate identities between all sequences. Semicolons mean strong similarity (>0.5 in the Gonnet PAM 250 matrix) and dots weak similarity (<0.5).
